# Efficacy of Horticultural Therapy on Positive, Negative, and Affective Symptoms in Individuals with Schizophrenia: A Systematic Review and Meta-Analysis of Randomized Controlled Trials

**DOI:** 10.3390/healthcare12212104

**Published:** 2024-10-22

**Authors:** Yi-Wen Lee, Tzu-Ting Chen, Chih-Wei Hsu, Ming-De Chen, Pao-Yen Lin, Yu-Chi Huang, Chi-Fa Hung, Chyi-Rong Chen

**Affiliations:** 1 Department of Nursing, Kaohsiung Chang Gung Memorial Hospital and Chang Gung University College of Medicine, Kaohsiung 833401, Taiwan; hikare2000@adm.cgmh.org.tw; 2 Department of Psychiatry, Kaohsiung Chang Gung Memorial Hospital and Chang Gung University College of Medicine, Kaohsiung 833401, Taiwan; tracy5824@cgmh.org.tw (T.-T.C.); py1029@cgmh.org.tw (P.-Y.L.); yuchihuang@cgmh.org.tw (Y.-C.H.); chifa@cgmh.org.tw (C.-F.H.); 3 Department of Occupational Therapy, Shu-Zen Junior College of Medicine and Management, Kaohsiung 821004, Taiwan; 4 Department of Occupational Therapy, College of Health Sciences, Kaohsiung Medical University, Kaohsiung 80708, Taiwan; mdchen@kmu.edu.tw

**Keywords:** schizophrenia, horticultural therapy, psychiatric symptoms, emotions, meta-analysis

## Abstract

**Background/Objectives:** Positive symptoms, negative symptoms, and emotional disturbances are core features of schizophrenia. Although horticultural therapy (HT) has shown promise as an adjunctive treatment, evidence supporting its effectiveness remains limited. This systematic review and meta-analysis aimed to assess the impact of HT on total symptoms, positive symptoms, negative symptoms, and emotional disturbances in individuals with schizophrenia. **Methods:** We conducted a search for randomized controlled trials (RCTs) published up to March 2024 across multiple databases, including PubMed, Embase, Cochrane Library, CINAHL, CEPS, CNKI, Wanfang, and Yiigle. A random-effects model was employed to calculate the standardized mean difference (SMD). **Results:** A total of 35 studies enrolling 2899 participants were included. Our results indicated that, in the short term (≦3 months), HT has moderate to large effect sizes on total symptoms (SMD = 0.690, 95% CI 0.463 to 0.916), positive symptoms (SMD = 0.695, 95% CI 0.038 to 1.351), negative symptoms (SMD = 0.681, 95% CI 0.395 to 0.967), depression (SMD = 0.646, 95% CI 0.334 to 0.959), and anxiety (SMD = 0.627, 95% CI 0.364 to 0.890), with more pronounced benefits for anxiety symptoms in patients with a shorter duration of illness. In the long term (>3 months), HT shows large effect sizes for total symptoms (SMD = 1.393, 95% CI 0.858 to 1.928), negative symptoms (SMD = 1.389, 95% CI 0.935 to 1.842), anxiety (SMD = 1.541, 95% CI 1.042 to 2.040), and moderate to large effect sizes for positive symptoms (SMD = 0.667, 95% CI 0.077 to 1.258) and depression (SMD = 0.707, 95% CI 0.198 to 1.217). Additionally, longer weekly treatment durations are associated with better outcomes for total symptoms and negative symptoms. Schizophrenia patients with more severe initial symptoms may be potential responders to HT. **Conclusions:** These findings support the efficacy of HT in improving symptoms and emotional well-being in schizophrenia patients. Further trials with more rigorous designs are warranted to confirm these benefits.

## 1. Introduction

Schizophrenia affects nearly 1% of the global population [1]. Core symptoms of schizophrenia include positive symptoms such as hallucinations, delusions, disorganized behavior, and incoherent speech, as well as negative symptoms such as lack of motivation, social withdrawal, and impaired verbal expression [2]. Emotional disturbances, including depression and anxiety, are also prevalent in individuals with schizophrenia [3]. Research has shown that these emotional issues are frequently associated with more severe psychiatric symptoms [4,5] and poorer treatment outcomes [6,7]. Furthermore, cognitive dysfunction is also a prevalent characteristic of schizophrenia, which leads to long-term disabilities [8,9]. The possible causes of these symptoms include the dopamine hypothesis of schizophrenia, which posits that overactivation of dopamine in the mesolimbic pathway leads to positive symptoms, while reduced dopamine levels in the mesocortical pathway and prefrontal cortex are linked to negative symptoms [10]. Recent studies have also suggested that low levels of brain-derived neurotrophic factor (BDNF) may be associated with emotional regulation and memory impairments in schizophrenia [11]. Effective management of schizophrenia requires addressing both psychiatric symptoms and associated emotional issues.

Currently, pharmacotherapy is the primary treatment for schizophrenia and has demonstrated substantial efficacy in alleviating positive symptoms, depression, and anxiety [10,12,13]. However, its effectiveness in alleviating negative symptoms remains limited [14]. Moreover, pharmacotherapy often comes with side effects such as tardive dyskinesia, restlessness, sedation, sexual dysfunction, and poor adherence to treatment [15,16]. Non-pharmacological interventions, such as physical exercise, have been explored as alternative treatments, showing promising preliminary results [17]. Nonetheless, approximately one-third of patients with schizophrenia may drop out of physical activity regimens [18]. Thus, there is a need to explore other non-pharmacological approaches to improve treatment for patients with schizophrenia.

Horticultural therapy (HT), as defined by the American Horticultural Therapy Association, involves engaging individuals in gardening-related activities facilitated by a trained therapist to achieve specific treatment goals [19]. Therapeutic horticulture, on the other hand, refers to programs involving plant-related activities aimed at improving well-being through active and passive engagement. These programs can be implemented by various healthcare providers [20]. Of note, the terms “horticultural therapy” and “therapeutic horticulture” are often used interchangeably [21]. Recent literature distinguishes between two types of HT: participatory HT, which includes activities such as cultivating, trimming, weeding, and floriculture, and observational HT, which involves garden tours and viewing natural scenery [22,23]. Both types of HT have long been used as therapeutic modalities. They can enhance participants’ physical, mental, and emotional well-being through interaction with or observation of horticultural activities involving fruits, vegetables, flowers, and plants [24,25].

Although the theories and mechanisms underlying HT are not yet conclusive, they generally fall into several categories: (1) The biophilia hypothesis suggests that humans have an innate tendency to connect with nature, leading to physiological and psychological benefits [26,27]. (2) Ulrich’s Stress Reduction Theory posits that natural stimuli activate the parasympathetic nervous system, facilitating psychophysiological stress recovery [28,29]. (3) Attention Restoration Theory suggests that horticultural activities, which involve interacting with nature, help shift attention away from negative emotions, reducing depression and anxiety [30]. (4) The setting for HT, often a healing or therapeutic garden, is designed to meet the basic human need for contact with nature, promoting a sense of security and physical comfort while maintaining a calm atmosphere to maximize therapeutic potential [21,31]. (5) Social interaction within HT programs offers significant benefits for individuals with schizophrenia, many of whom experience social isolation. Engaging in therapeutic garden activities can provide opportunities for social interaction and network building, thus enhancing overall social well-being [21]. (6) Horticultural tasks such as planting, potting, and lawn mowing are considered light to moderate physical activities [21,32]. Therefore, the benefits of physical activity for schizophrenia might be obtained through these gardening activities.

Previous meta-analyses have reported mixed results regarding the efficacy of HT for schizophrenia. One meta-analysis, including only one randomized controlled trial (RCT), found that horticultural activities benefited emotional outcomes but had no significant effect on well-being [21]. However, this study’s conclusions were limited by the inclusion of only one trial [21]. A subsequent review, which included five studies on HT for schizophrenia, found potential benefits in social, vocational, psychological, and neuropsychological functioning [33]. However, as this article did not employ a systematic review methodology and included only one randomized controlled trial, its findings remain inconclusive [33]. A more recent meta-analysis that incorporated both RCTs and non-RCTs supported the efficacy of HT in improving psychiatric symptoms and quality of life for individuals with schizophrenia [34]. Additionally, horticultural therapy significantly enhanced rehabilitation outcomes, though they showed no significant effects on social functioning [34]. The primary limitation of the study mentioned was the inclusion of non-randomized controlled trials [34], which may have increased the risk of bias [35]. Furthermore, this analysis did not examine positive symptoms, negative symptoms, or emotional states in individuals with schizophrenia [34]. Another meta-analysis of 18 RCTs found moderate benefits of HT on mental health but included diverse populations, such as individuals with mental disorders, elderly people, and those with stroke or dementia [30]. In summary, the limitations of existing studies, including non-randomized controlled trials, diverse populations, and unclear effects on various psychiatric symptoms, highlight the need for more rigorous research to fully understand the effects of HT on positive symptoms, negative symptoms, and emotional states in individuals with schizophrenia.

The optimal dosage for HT has yet to be determined. Previous meta-analyses have noted that only a few hours of horticultural activities can help alleviate symptoms of depression and anxiety, while several weeks to months of HT may benefit physical health [36]. However, the subjects of those studies were not individuals with schizophrenia. Recent studies have indicated that 100 to 500 min of horticultural activities are most effective for stress management across various populations [37]. Additionally, research has shown that a 4- to 8-week intervention period yields better outcomes for older adults with depressive symptoms [22]. Research also suggests that engaging in 1 to 2 h of horticultural therapy per week over a period of 1.5 to 12 months can enhance the quality of life and physical function in older adults [38]. However, the appropriate treatment regimen of horticultural activities for individuals with schizophrenia remains to be further investigated.

Although HT shows promise, existing meta-analyses have not thoroughly investigated its effectiveness in patients with schizophrenia. Therefore, this study aimed to (1) systematically review RCTs to assess the impact of HT on symptoms and emotional states in individuals with schizophrenia and (2) identify moderators of HT effectiveness by examining factors such as sample characteristics and intervention variables.

## 2. Materials and Methods

### 2.1. Search Strategy

This systematic review adhered to the Preferred Reporting Items for Systematic Reviews and Meta-Analyses (PRISMA) guidelines [39]. Literature searches were independently conducted by two researchers (Y.-W.L. and C.-R.C.) across several databases: Pubmed, Embase, Cochrane Library, CINAHL, and Chinese databases including Airiti Library (CEPS), China National Knowledge Infrastructure (CNKI), Wanfang database, and Yiigle. These databases are commonly used in systematic reviews regarding non-pharmacological interventions for individuals with schizophrenia [40]. The search was conducted from inception to 31 March 2024. Additionally, a manual search for relevant studies was performed in review articles and reference lists of included studies.

Keywords used for the literature search included schizophrenia, severe mental illness, horticultural therapy, gardening, planting, and farming. Corresponding Chinese keywords were also applied. Boolean operators and filters were employed to refine the search. Detailed search strings and databases used are listed in Table S1.

### 2.2. Inclusion and Exclusion Criteria

Inclusion criteria were as follows: (1) peer-reviewed journal articles utilizing RCT design; (2) inclusion of adult individuals (age ≥ 18) diagnosed with schizophrenia; (3) interventions involving therapeutic activities such as gardening, plant cultivation, or other forms of HT; and (4) assessment of outcomes including symptoms and emotional states. Exclusion criteria were (1) studies with irrelevant outcomes and (2) dissertations or conference proceedings. There were no language restrictions. The literature selection was conducted independently by two researchers (Y.-W.L. and T.-T.C.), with discrepancies resolved through discussions with the corresponding author to reach a consensus.

### 2.3. Data Extraction

Two independent researchers (Y.-W.L. and T.-T.C.) used a predefined data extraction form to collect study information, including author names, publication years, demographic characteristics (e.g., age, gender), and study design variables (e.g., intervention frequency, duration). Discrepancies were resolved through discussion to reach a consensus. Data on total symptoms, positive symptoms, negative symptoms, depression, and anxiety in individuals with schizophrenia were extracted. Recent meta-analyses have identified that the most common duration for non-pharmacological interventions in individuals with schizophrenia is 12 weeks [17,41]. Additionally, some studies suggest that a minimum 12-week intervention period should be considered as a benchmark for the effectiveness of schizophrenia treatments [42]. Therefore, pre-test and post-test data were categorized into two-time frames for analysis: short-term effects (≦3 months) and long-term effects (>3 months).

### 2.4. Quality Assessment of the Literature

The quality of the literature was assessed using the Cochrane Risk of Bias (RoB) tool, which addresses selection bias, allocation concealment, performance bias, detection bias, attrition bias, publication bias, and other potential biases [43]. Revman 5.4 software was used to generate the risk of bias graph and summary table. The assessment was independently performed by two researchers (Y.-W.L. and T.-T.C.), with discrepancies resolved through discussion with the corresponding author to achieve consensus.

### 2.5. Meta-Analysis, Meta-Regression, and Subgroup Analysis

Meta-analysis was conducted using the mean and standard deviations of change scores between pre-test and post-test assessments [43]. Given potential heterogeneity across studies, a random-effects model was used to calculate standardized mean differences (SMDs) and 95% confidence intervals (95% CI) for each outcome. SMD, also known as Cohen’s d [44], was interpreted based on Cohen’s criteria: 0.2 for small, 0.5 for moderate, and 0.8 for large effect sizes [45]. Meta-regressions were performed to examine the impact of participant characteristics (e.g., mean age, percentage of female participants, duration of illness) and intervention details (e.g., weekly intervention duration). In research on non-pharmacological treatments for schizophrenia, these variables have been identified as moderators of treatment effectiveness [40,46]. The subgroup analysis was conducted based on participants’ initial severity of symptoms, with groups classified according to the mean total scores on the Positive and Negative Symptoms Scale (PANSS) or Brief Psychiatric Rating Scale (BPRS). Disease severity criteria were based on the definitions provided by Leucht et al. [47,48]: a PANSS score of 58 or BPRS score of 31 indicated “mildly ill”, a PANSS score of 75 or BPRS score of 41 indicated “moderately ill”, and a PANSS score of 116 or BPRS score of 53 indicated “severely ill”. Sensitivity analysis involved systematically excluding individual studies to assess their impact on overall meta-analysis results. Heterogeneity was assessed using *I^2^* statistics, with values of 25%, 50%, and 75% indicating low, moderate, and high heterogeneity, respectively. Publication bias was evaluated through visual inspection of funnel plots and Egger’s test. All statistical analyses were conducted using Comprehensive Meta-Analysis Version 4 software (Biostat Inc., Englewood, NJ, USA). This study was registered in INPLASY (INPLASY202440073).

### 2.6. Certainty of Evidence

The certainty of evidence was evaluated using the Grading of Recommendations, Assessment, Development, and Evaluations (GRADE) framework [49]. This approach assessed evidence certainty from RCTs by examining the risk of bias, inconsistency, indirectness, imprecision, and publication bias.

## 3. Results

### 3.1. Search Results and Study Characteristics

**Figure 1:**
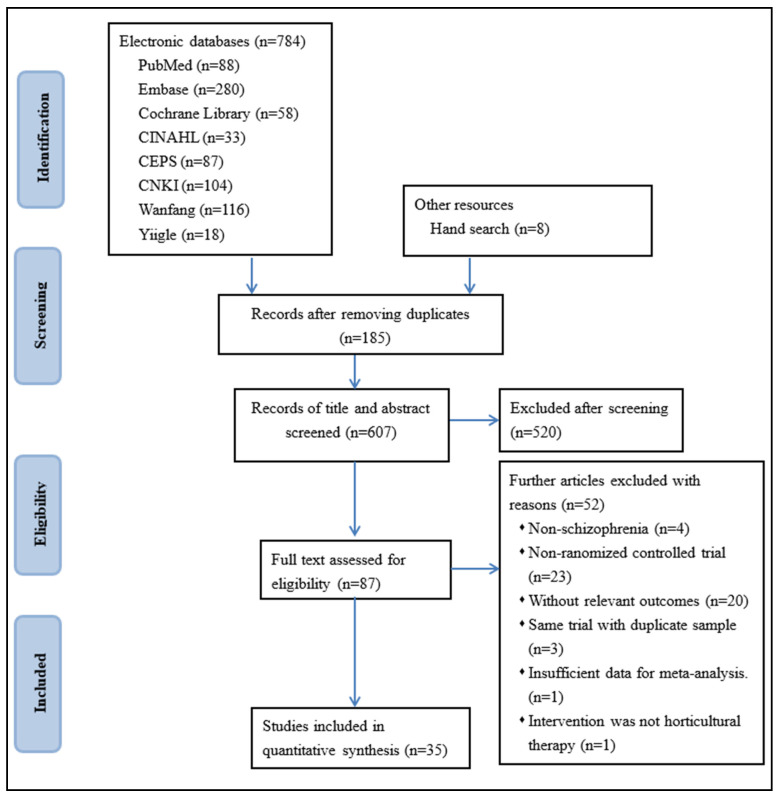
PRISMA flow diagram.

A total of 792 records were identified through the search process. After removing duplicates and screening abstracts, 87 articles were selected for full-text review. Of these, 52 studies were further excluded. One study was excluded due to insufficient data, as the authors did not report the duration of the intervention [50], and we were unable to contact the corresponding author. After team discussions, it was decided that the study lacked sufficient information for analysis [50]. Therefore, a total of 35 full-text articles were included in the analysis. These studies all reported on total symptoms, positive symptoms, negative symptoms, depression, or anxiety in individuals with schizophrenia. The search process is detailed in Figure 1. The included studies were published between 2001 and 2024. In total, 2899 participants were involved, with 1457 assigned to the HT group and 1442 to the control group. Of the participants, 44.68% were female. The mean age of the participants was 43.86 years (range: 30.17 to 61.68 years), with an average disease duration of 13.35 years (range: 2.45 to 36.04 years).

Among the included studies, 30 studies were conducted in China [51,52,53,54,55,56,57,58,59,60,61,62,63,64,65,66,67,68,69,70,71,72,73,74,75,76,77,78,79,80], two in Hong Kong [81,82], two in Taiwan [83,84], and one in Japan [85]. Five studies were published in English [57,78,81,82,85], while the remaining studies were published in Mandarin. The studies in published Mandarin were retrieved from Chinese databases such as CEPS, CNKI, Wanfang database, and Yiigle. Notably, two articles by Chen (2023) [55] and Chen (2023) [54] both pertain to the same clinical trial. In the current study, Chen (2023) [55] was included in the analysis of symptoms, while Chen (2023) [54] was included in the analysis of depression. Regarding intervention characteristics, the average number of sessions per week was 3.2 (range: 1 to 7 sessions), with an average duration of 87.96 min per session (range: 30 to 210 min). The overall average intervention duration was 24.50 weeks (range: 2 to 102 weeks). All studies employed a group format and utilized participatory horticultural therapy, incorporating horticulture-related activities such as plant cultivation, bonsai creation, horticultural crafts, cooking, and sensory stimulation. The most commonly used horticultural activity was plant cultivation, including vegetables, fruits, and flowers. Some studies also combined participatory horticultural activities with observational activities, such as observation of plants and landscape viewing. Details of the included studies are presented in Table S2.

### 3.2. Risk of Bias in Individual Studies

**Figure 2:**
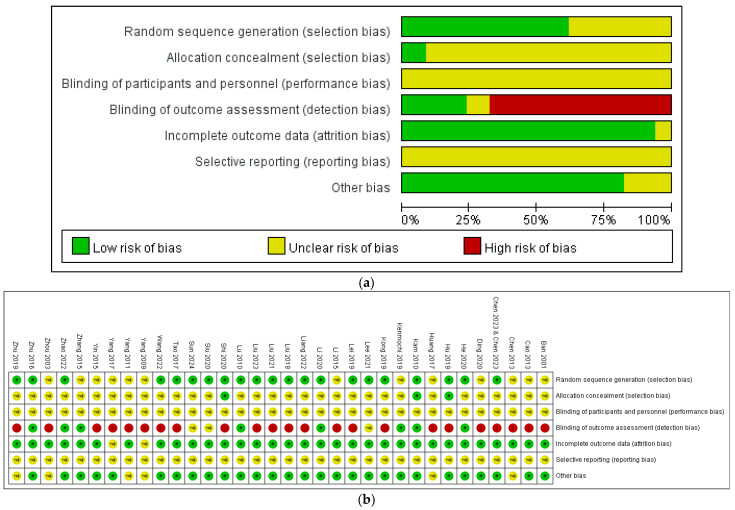
(**a**) Risk of bias graph. (**b**) Risk of bias summary for the included studies. Ban 2001 is the Ref. [51]; Cao 2013 is the Ref. [52]; Chen 2013 is the Ref. [53]; Chen 2023 and Chen 2023 are the Ref [54,55]; Ding 2020 is the Ref. [56]; He 2020 is the Ref. [57]; Hu 2019 is the Ref. [58]; Huang 2017 is the Ref. [59]; Kam 2010 is the Ref. [82]; Kenmochi 2019 is the Ref. [85]; Kong 2019 is the Ref. [60]; Lee 2021 is the Ref. [84]; Lei 2019 is the Ref. [61]; Li 2015 is the Ref. [62]; Li 2020 is the Ref. [63]; Liang 2022 is the Ref. [64]; Liu 2018 is the Ref. [67]; Liu 2021 is the Ref. [66]; Liu 2023 is the Ref. [65]; Lu 2010 is the Ref. [68]; Shi 2020 is the Ref. [69]; Siu 2020 is the Ref. [81]; Sun 2024 is the Ref. [80]; Tao 2017 is the Ref. [70]; Wang 2022 is the Ref. [71]; Yang 2009 is the Ref. [73]; Yang 2011 is the Ref. [72]; Yang 2017 is the Ref. [83]; Yin 2015 is the Ref. [74]; Zhang 2015 is the Ref. [75]; Zhao 2022 is the Ref. [76]; Zhou 2003 is the Ref. [77]; Zhu 2016 is the Ref. [78]; Zhu 2019 is the Ref. [79].

Thirteen trials were rated as unclear due to insufficient descriptions of the random allocation methods, while 21 trials were rated as low risk. For allocation concealment, 31 trials were rated as unclear due to insufficient descriptions of the methods, and three trials were rated as low risk. Regarding blinded outcome assessment, 23 trials were classified as high risk, three trials as unclear, and eight trials provided detailed descriptions of assessor blinding, resulting in a low-risk rating. All trials were rated as unclear regarding the blinding of participants and personnel. In terms of incomplete outcome reporting, two trials lacked clear explanations regarding participant withdrawals and were rated as unclear, whereas 32 trials were rated as low risk. All trials were rated as unclear for selective reporting due to the lack of trial registration. Lastly, in the category of other bias, six trials were rated as unclear, and 28 trials were rated as low risk. The risk of bias assessment is presented in Figure 2.

### 3.3. Synthesis of Results

#### 3.3.1. Total Symptoms

**Figure 3:**
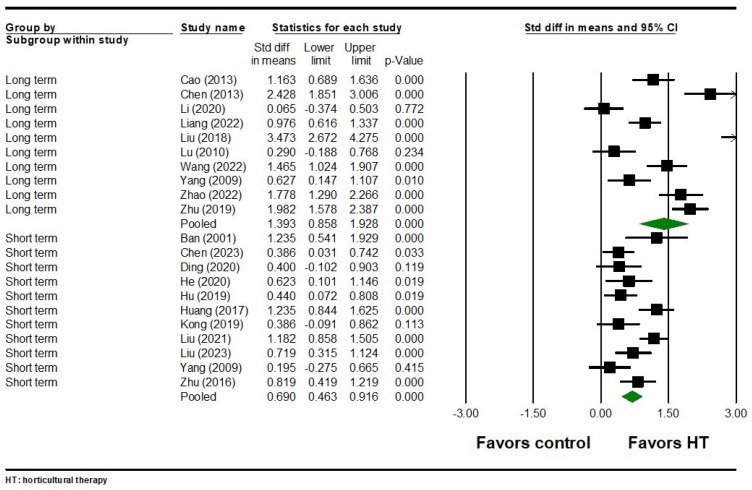
Forest plot of HT on total symptoms in individuals with schizophrenia. Ban 2001 is the Ref. [51]; Cao 2013 is the Ref. [52]; Chen 2013 is the Ref. [53]; Chen 2023 is the Ref [55]; Ding 2020 is the Ref. [56]; He 2020 is the Ref. [57]; Hu 2019 is the Ref. [58]; Huang 2017 is the Ref. [59]; Kong 2019 is the Ref. [60]; Li 2020 is the Ref. [63]; Liang 2022 is the Ref. [64]; Liu 2018 is the Ref. [67]; Liu 2021 is the Ref. [66]; Liu 2023 is the Ref. [65]; Lu 2010 is the Ref. [68]; Wang 2022 is the Ref. [71]; Yang 2009 is the Ref. [73]; Zhao 2022 is the Ref. [76]; Zhu 2016 is the Ref. [78]; Zhu 2019 is the Ref. [79].

For total symptoms, 11 studies involving 1,034 participants revealed that HT had a significant short-term effect (SMD = 0.690, 95% CI = 0.463 to 0.916, *p* < 0.001) (Figure 3). There was significant heterogeneity among the included studies (Q = 30.765, I^2^ = 67.495, *p* = 0.001). In the long term, ten studies involving 900 participants also showed that HT had a significant effect on total symptoms (SMD = 1.393, 95% CI = 0.858 to 1.928, *p* < 0.001) (Figure 3). Heterogeneity was significant in these studies as well (Q = 115.877, I^2^ = 92.233, *p* < 0.001). No publication bias was detected for either short-term or long-term effects on total symptoms (Egger’s test *p* were 0.605 and 0.134, respectively).

#### 3.3.2. Positive Symptoms

**Figure 4:**
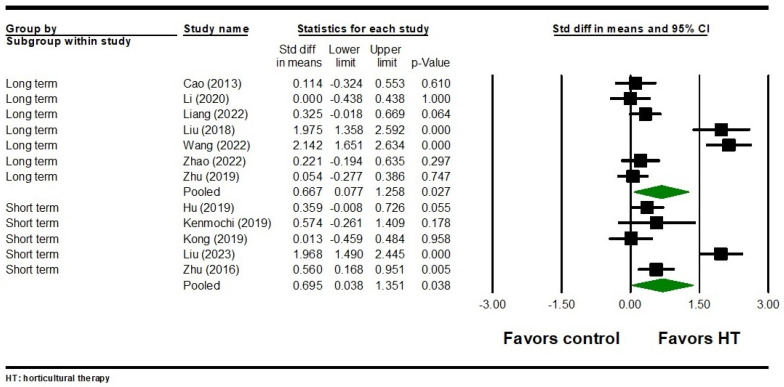
Forest plot of HT on positive symptoms in individuals with schizophrenia. Cao 2013 is the Ref. [52]; Hu 2019 is the Ref. [58]; Kenmochi 2019 is the Ref. [85]; Kong 2019 is the Ref. [60]; Li 2020 is the Ref. [63]; Liang 2022 is the Ref. [64]; Liu 2018 is the Ref. [67]; Liu 2023 is the Ref. [65]; Wang 2022 is the Ref. [71]; Zhao 2022 is the Ref. [76]; Zhu 2016 is the Ref. [78]; Zhu 2019 is the Ref. [79].

For positive symptoms, five studies with 412 participants found that HT had a significant short-term effect with notable heterogeneity (SMD = 0.695, 95% CI = 0.038 to 1.351, *p* = 0.038; Q = 38.912, I^2^ = 89.720, *p* < 0.001) (Figure 4). Seven studies involving 682 participants revealed a significant long-term effect of HT on positive symptoms, with substantial heterogeneity (SMD = 0.667, 95% CI = 0.077 to 1.258, *p* = 0.027; Q = 82.328, I^2^ = 92.712, *p* < 0.001) (Figure 4). There was no significant publication bias in either the short-term effect (Egger’s test *p* = 0.801) or the long-term effect on positive symptoms (Egger’s test *p* = 0.070).

#### 3.3.3. Negative Symptoms

**Figure 5:**
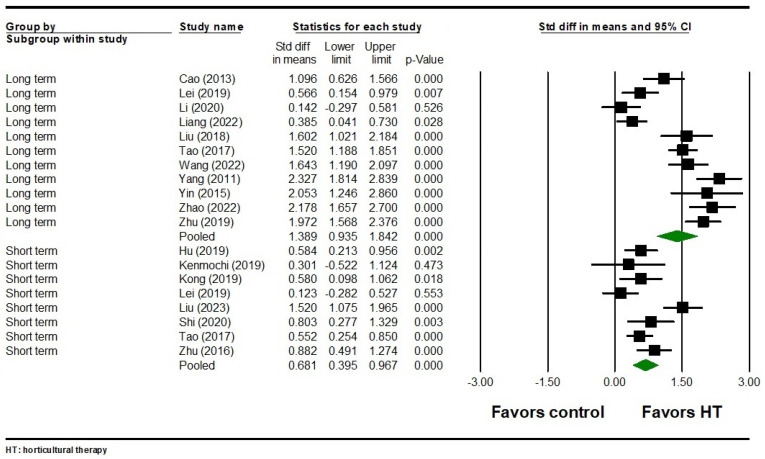
Forest plot of HT on negative symptoms in individuals with schizophrenia. Cao 2013 is the Ref. [52]; Hu 2019 is the Ref. [58]; Kenmochi 2019 is the Ref. [85]; Kong 2019 is the Ref. [60]; Lei 2019 is the Ref. [61]; Li 2020 is the Ref. [63]; Liang 2022 is the Ref. [64]; Liu 2018 is the Ref. [67]; Liu 2023 is the Ref. [65]; Shi 2020 is the Ref. [69]; Tao 2017 is the Ref. [70]; Wang 2022 is the Ref. [71]; Yang 2011 is the Ref. [72]; Yin 2015 is the Ref. [74]; Zhao 2022 is the Ref. [76]; Zhu 2016 is the Ref. [78]; Zhu 2019 is the Ref. [79].

Across eight studies involving 752 participants, HT was found to have a significant short-term effect on negative symptoms (SMD = 0.681, 95% CI = 0.395 to 0.967, *p* < 0.001) (Figure 5). Significant heterogeneity was observed among the included studies (Q = 24.124, I^2^ = 70.984, *p* = 0.001). Regarding the long-term effects of HT on negative symptoms, 11 studies involving 1,090 participants showed a significant effect with substantial heterogeneity (SMD = 1.389, 95% CI = 0.935 to 1.842, *p* < 0.001; Q = 111.670, I^2^ = 91.045, *p* < 0.001) (Figure 5). Egger’s test indicated no publication bias for both short-term (*p* = 0.873) and long-term (*p* = 0.210) effects on negative symptoms.

#### 3.3.4. Emotional Outcomes

**Figure 6:**
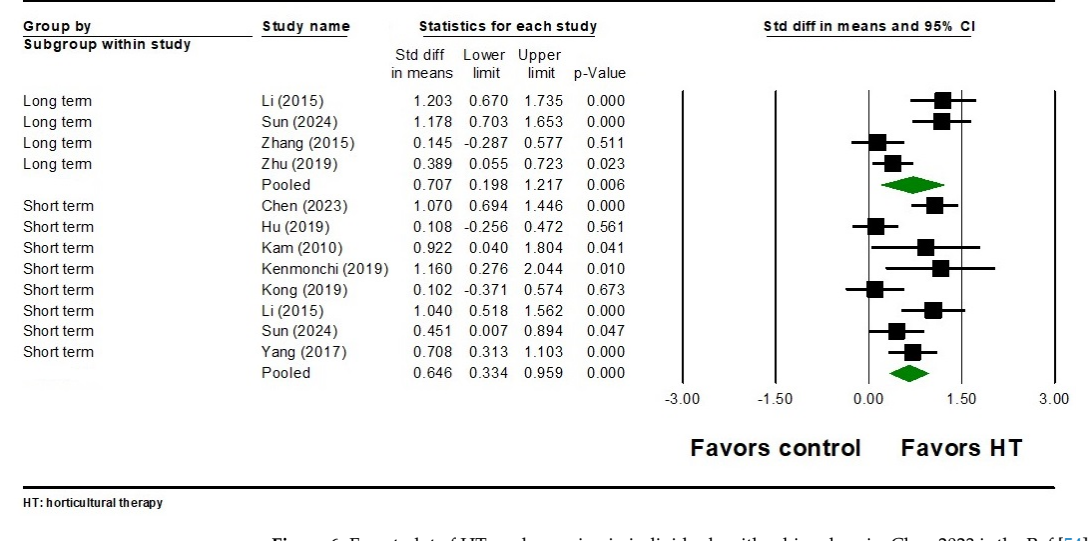
Forest plot of HT on depression in individuals with schizophrenia. Chen 2023 is the Ref [54]; Hu 2019 is the Ref. [58]; Kam 2010 is the Ref. [82]; Kenmochi 2019 is the Ref. [85]; Kong 2019 is the Ref. [60]; Li 2015 is the Ref. [62]; Sun 2024 is the Ref. [80]; Yang 2017 is the Ref. [83]; Zhang 2015 is the Ref. [75]; Zhu 2019 is the Ref. [79].

**Figure 7:**
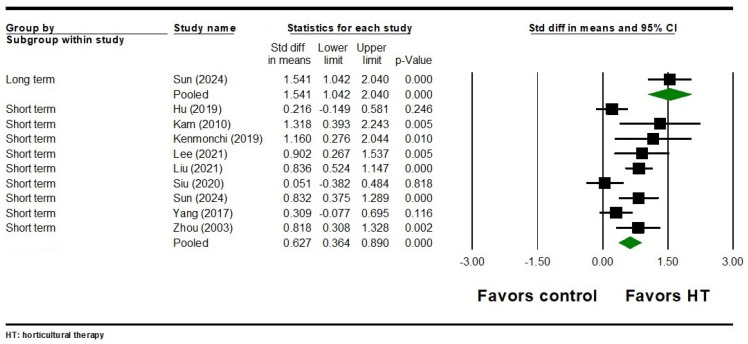
Forest plot of HT on anxiety in individuals with schizophrenia. Hu 2019 is the Ref. [58]; Kam 2010 is the Ref. [82]; Kenmochi 2019 is the Ref. [85]; Lee 2021 is the Ref. [84]; Liu 2021 is the Ref. [66]; Siu 2020 is the Ref. [81]; Sun 2024 is the Ref. [80]; Yang 2017 is the Ref. [83]; Zhou 2003 is the Ref. [77].

For emotional outcomes, eight studies involving 603 participants found that HT had a significant short-term effect on depression (SMD = 0.646, 95% CI = 0.334 to 0.959, *p* < 0.001) (Figure 6). Significant heterogeneity was observed among the included studies (Q = 22.777, I^2^ = 69.268, *p* = 0.002). In terms of the long-term effects of HT on depression, four studies involving 367 participants showed a significant effect with heterogeneity (SMD = 0.707, 95% CI = 0.198 to 1.217, *p* = 0.006; Q = 16.373, I^2^ = 81.678, *p* = 0.001) (Figure 6). Additionally, night studies involving 710 participants found a significant short-term effect of HT on anxiety symptoms (SMD = 0.627, 95% CI = 0.364 to 0.890, *p* < 0.001) (Figure 7). Significant heterogeneity was noted among these studies (Q = 21.125, I^2^ = 62.130, *p* = 0.007). As for the long-term effects of HT on anxiety, one study involving 80 participants found a significant effect (SMD = 1.541, 95% CI = 1.042 to 2.040, *p* < 0.001) (Figure 7). Egger’s test did not reveal significant publication bias for either the short-term or long-term emotional outcomes (all Egger’s *p* > 0.05).

The results of the meta-analysis are summarized in Table 1, while the funnel plots of each comparison are presented in Figures S1–S9. The sensitivity analysis showed no change in the overall results after sequentially removing the included studies.

### 3.4. Meta-Regression and Subgroup Analysis

Meta-regressions were conducted to examine the short-term and long-term effects on total symptoms, positive symptoms, negative symptoms, depression, and anxiety; variables assessed included mean age, percentage of female participants, duration of illness, and weekly amount of intervention in minutes. For short-term effects, the results indicated that the duration of illness was inversely correlated with treatment effects (*β*=−0.036, *p* = 0.005). No other intervention or sample characteristics were significantly associated with short-term effects (all *p* > 0.05). Regarding long-term effects, a greater amount of weekly intervention was associated with a greater improvement in total symptoms (*β* = 0.004, *p* = 0.031) and negative symptoms (*β* = 0.004, *p* = 0.018). No other significant predictors were identified for the long-term effects of HT on patients with schizophrenia (all *p* > 0.05). The results of the meta-regression are presented in Table S3.

The subgroup analysis was based on initial severity of symptoms. In this meta-analysis, Participants were divided into “mildly illness”, “moderately illness”, or “severely illness” subgroups according to the mean scores of PANSS and BPRS. The detailed results of the subgroup analysis are presented in Table S4.

In the short-term, the results indicated that HT had a significant effect on total symptoms, positive symptoms, and negative symptoms among participants with mild illness (SMDs were 0.689, 0.360, and 0.661 for the above outcomes, respectively, all ps < 0.05). There was no significant effect on depression and anxiety among participants in the mild illness subgroup (ps were 0.202 and 0.197, respectively). As for the moderately illness subgroup, HT had significant improvement in total symptoms and positive symptoms (SMDs were 0.594 and 1.968, respectively, and both *p* < 0.001). There were no studies that investigated the short-term effects of HT on negative symptoms and emotional outcomes in this study. With respect to the severely illness subgroup, significant effects of HT were found in total symptoms, negative symptoms, depression, and anxiety (SMDs were 0.787, 1.520, 1.070, and 0.836 for the above outcomes, respectively, all *p* < 0.05). There were no studies that investigated the effects of HT on positive symptoms in this subgroup.

In terms of long-term effects, no study was categorized as a severe illness subgroup. The results indicated that HT had no significant effects on total symptoms, positive symptoms, negative symptoms, and depression among participants in the mild illness subgroup (ps were 0.069, 0.449, 0.257 and 0.511, respectively). However, when it comes to the moderately illness subgroup, HT had significant improvement in total symptoms, positive symptoms, negative symptoms, and depression (SMD were 1.858, 0.900, 1.331, and 0.389 for the above outcomes, respectively, all *p* < 0.05). There was insufficient data on the long-term effect of HT on anxiety for subgroup analysis.

### 3.5. Certainty of Evidence

According to the GRADE system, the certainty of evidence for the short-term effects on positive symptoms, long-term effects of depression, and long-term effects of anxiety were rated as “very low” due to risks of bias, high heterogeneity, and imprecision in effect estimates. The certainty of evidence for all other outcomes was rated as “low” due to risks of bias and high heterogeneity. The certainty of evidence based on the GRADE system is presented in Table S5.

## 4. Discussion

This systematic review and meta-analysis examined the effects of HT on symptoms and emotional states in individuals with schizophrenia. The meta-analysis revealed that short-term interventions (lasting less than three months) significantly improved total symptoms, positive symptoms, negative symptoms, depression, and anxiety. Meta-regression indicated that individuals with a shorter duration of illness experienced greater reductions in anxiety through HT. For interventions lasting longer than three months, significant improvements were observed in total symptoms, positive symptoms, negative symptoms, depression, and anxiety. Meta-regression revealed that longer weekly treatment times of HT were associated with better outcomes for total symptoms and negative symptoms. Moreover, our findings indicate that longer durations of horticultural therapy enhance therapeutic efficacy, and individuals with higher baseline symptom severity may be optimal candidates. However, due to risks of bias, inconsistency, and imprecision in the included studies, the certainty of evidence for these findings ranges from low to very low. Additionally, all studies included in this review were conducted in Asia. Thus, these results should be interpreted with caution, and future clinical trials from other areas are necessary to validate these findings.

Our study found that HT has a moderate to large short-term effect (SMD = 0.690) and a large long-term effect (SMD = 1.393) on total symptoms in individuals with schizophrenia. These results indicate that HT showed greater improvement in total symptoms compared with the control group, with a mean score of 0.690 standard deviations higher for treatments lasting around three months and 1.393 standard deviations for treatments extending beyond three months. These results are consistent with previous research, though the effect size in our study was smaller compared with earlier studies that included both RCTs and non-RCTs [34]. Our exclusive focus on RCTs, which involved a larger sample size, allowed for a more comprehensive analysis of both short-term and long-term benefits. Moreover, the subgroup analysis also indicated that HT has greater effectiveness for individuals with more severe initial symptoms. The findings support the effectiveness of horticultural therapy activities in improving total symptoms in individuals with schizophrenia. Recent studies have explored the benefits of HT as a form of light to moderate intensity physical activities on brain health, finding that horticultural activities like soil digging or crop planting can enhance neurotransmitters (e.g., serotonin, dopamine) and regulate inflammatory cytokines in adults [86]. Thus, the neurobiological mechanisms of horticultural activities for individuals with schizophrenia warrant further investigation in future research.

For negative symptoms, HT demonstrated moderate to large short-term effects (SMD = 0.681) and large long-term effects (SMD = 1.389). HT demonstrated greater improvement in negative symptoms compared with the control group, with a mean score of 0.681 standard deviations higher for treatments lasting three months and 1.389 standard deviations for treatments extending beyond three months. Meta-regression results indicated that longer weekly durations of HT activities were positively correlated with better treatment outcomes. This aligns with previous research suggesting that higher doses of HT are associated with better therapeutic outcomes [87]. The nature of horticultural activities, which require daily attention to plants, and extended intervals between therapeutic sessions may affect the engagement of participants with natural elements, thereby influencing the efficacy of the therapy. Moreover, the subgroup analysis found that individuals with more severe initial symptoms experienced greater improvement in negative symptoms with HT. In summary, increased weekly therapy time, longer treatment durations, and more severe initial symptoms of participants may serve as moderators for HT in improving negative symptoms in individuals with schizophrenia.

Regarding positive symptoms, our study found moderate to large effects of HT, both in the short term (SMD = 0.695) and long term (SMD = 0.667). HT demonstrated an average improvement in positive symptoms of 0.667 to 0.695 standard deviations higher than the control group. Additionally, the results of the subgroup analysis consistently showed smaller effect sizes of HT for individuals with mild initial severity and larger effect sizes for those with moderate initial severity. Previous research has found that horticultural activities can encourage individuals with schizophrenia to engage in outdoor activities and increase their level of physical activity [26,36]. Moreover, several studies incorporated horticultural craft-making in the programs [52,56,57,58]. Evidence has supported that physical activity and craft-making benefit the positive symptoms of schizophrenia [18,40]. Prior studies have reported that horticultural activities can divert attention from symptoms, foster a sense of productivity [88], and enhance self-esteem [89], supporting their role as an adjunctive therapy for schizophrenia.

HT also demonstrated a moderate to large effect on depressive symptoms, both in the short term (SMD = 0.646) and long term (SMD = 0.707). These findings suggest that horticultural therapy provides an improvement in depressive symptoms for individuals with schizophrenia, with an average effect ranging from 0.646 to 0.707 standard deviations higher than the control group. Previous research supports the benefits of HT for depressive symptoms in older adults [22,90], and our findings are consistent with these studies. Prior meta-analyses have indicated significant effects on emotional states, and our study, which encompassed clinical trials and studies with large sample sizes, is consistent with this research [21]. The results of subgroup analysis also indicate that in terms of short-term effects, HT leads to greater improvements in depression among participants with more severe initial symptoms. Recent studies also suggest that horticultural activities may improve BDNF levels in schizophrenia [91], enhancing self-regulation skills and providing opportunities for mindfulness among participants [92], thereby further contributing to the alleviation of depressive symptoms.

For anxiety symptoms, our study found that HT had a moderate to large effect (SMD = 0.627) within a three-month period. The effect was improved to a larger magnitude (SMD = 1.541) for HT programs lasting more than 3 months. These results showed that horticultural therapy improved anxiety symptoms in schizophrenia more than the control group, with short-term effects 0.627 and long-term effects 1.541 standard deviations higher. Meta-regression indicated that HT was particularly beneficial for individuals with a shorter duration of illness in the short term. The subgroup analysis of short-term effects indicates that HT had a significant effect on anxiety among the subgroup with more severe initial symptoms, while there was no significant effect observed in the subgroup with milder symptoms. Previous research has shown that horticultural activities can reduce parasympathetic nervous system activity [93], potentially facilitating relaxation. However, there was only one trial in this study regarding the long-term effect on anxiety, indicating a need for further research in this area.

All studies included in our review were randomized clinical trials conducted in Asia, where HT has been widely adopted [87]. In Chinese culture, philosophies such as Confucianism and Taoism emphasize the harmonious relationship between nature and humans for the promotion of overall well-being [94,95]. This cultural context may facilitate the implementation of HT in Asia. Moreover, HT programs in Asia are often designed to include both outdoor and indoor activities [87]. Common outdoor activities involve planting vegetables and fruits, while indoor activities frequently include horticultural crafts and bonsai creation. In this study, most HT interventions in mental health care included both types of activities. Outdoor planting of fruits and vegetables requires extended growth periods and regular maintenance, such as watering and weeding, which helps increase physical activity levels. Indoor activities, on the other hand, require less space and are less affected by weather conditions, factors that likely contribute to the widespread adoption of HT in Asia. However, A recent meta-analysis examining the effectiveness of HT on stress found variations in outcomes across different ethnic groups [37]. Therefore, further investigation is required to determine whether our findings can be generalized to other regions.

Overall, this study includes more clinical trials compared with previous research [34]. It distinguishes between positive symptoms, negative symptoms, depression, and anxiety for analysis. Additionally, it differentiates between the short-term and long-term effects of horticultural therapy, thereby filling gaps in the existing knowledge. However, there are several limitations: (1) Most included studies lacked clinical trial registration and exhibited risks of bias, affecting the certainty of the evidence. (2) This study did not explore other functional aspects of HT, such as cognitive function, physical function, and quality of life. (3) All included trials were conducted in Asia, limiting the generalizability of the findings to other cultural contexts. (4) This study involved a wide range of horticultural activities, making it challenging to analyze and determine which specific activities are most effective. Future research should include more geographically diverse samples and different ethnic backgrounds, ensure proper clinical trial registration, and use rigorous study designs. Additionally, future studies should investigate the effects of HT on cognitive function, physical function, and quality of life in individuals with schizophrenia, particularly by incorporating biomarkers to rigorously validate its efficacy.

## 5. Conclusions

This meta-analysis supports the short-term efficacy of HT for improving total symptoms, positive symptoms, negative symptoms, depression, and anxiety in individuals with schizophrenia. It also suggests that a shorter duration of illness is associated with better reductions in anxiety symptoms through HT. In terms of long-term effects, HT shows significant benefits for total symptoms, positive symptoms, negative symptoms, depression, and anxiety. Additionally, longer weekly minutes of HT intervention correlate with greater improvements in total symptoms and negative symptoms. These findings provide preliminary evidence that HT could be a useful complementary therapy in patients with schizophrenia. The findings indicated that individuals with schizophrenia who exhibit more severe initial symptoms may be considered potential responders to HT and that extended durations of HT may lead to more substantial outcomes.

## Figures and Tables

**Table 1:** Short-term and long-term effects of HT on symptoms and emotions in individuals with schizophrenia.

			Effect Size			Heterogeneity		
	Studies	Total n	SMD	95% CI	*p* Value	*Q* Value	*p* Value	*I*^2^ %
**Short-term effect (≤3 months)**								
Total symptoms	11	1034	0.690	0.463–0.916	<0.001	30.765	0.001	67.495
Positive symptoms	5	412	0.695	0.038–1.351	0.038	38.912	<0.001	89.720
Negative symptoms	8	752	0.681	0.395–0.967	<0.001	24.124	0.001	70.984
Depression	8	603	0.646	0.334–0.959	<0.001	22.777	0.002	69.268
Anxiety	9	710	0.627	0.364–0.890	<0.001	21.125	0.007	62.130
**Long-term effect (>3 months)**								
Total symptoms	10	900	1.393	0.858–1.928	<0.001	115.877	<0.001	92.233
Positive symptoms	7	682	0.667	0.077–1.258	0.027	82.328	<0.001	92.712
Negative symptoms	11	1090	1.389	0.935–1.842	<0.001	111.670	<0.001	91.045
Depression	4	367	0.707	0.198–1.217	0.006	16.373	0.001	81.678
Anxiety	1	80	1.541	1.042–2.040	<0.001	0.000	>0.999	0.000

## Data Availability

The raw data supporting the conclusions of this article will be made available by the authors on request.

## Supplementary Materials

The following supporting information can be downloaded at https://www.mdpi.com/article/10.3390/healthcare12212104/s1, Table S1. Search texts and strategies. Table S2. Details of included studies. Table S3. Meta-regression for examining moderator relationships. Table S4. Subgroup analysis based on initial severity of symptoms. Table S5. GRADE summary table. Figure S1. Funnel plot of total symptom (short term effect). Figure S2. Funnel plot of total symptom (long term effect). Figure S3. Funnel plot of positive symptoms (short term effect). Figure S4. Funnel plot of positive symptoms (long term effect). Figure S5. Funnel plot of negative symptoms (short term effect). Figure S6. Funnel plot of negative symptoms (long term effect). Figure S7. Funnel plot of Depression (short term effect). Figure S8. Funnel plot of Depression (long term effect). Figure S9. Funnel plot of anxiety (short term effect).
