# Supplementary material for: Efficacy of Horticultural Therapy on Positive, Negative, and Affective Symptoms in Individuals with Schizophrenia: A Systematic Review and Meta-Analysis of Randomized Controlled Trials
Source: Healthcare (Basel). 2024 Oct 22;12(21):2104. doi: 10.3390/healthcare12212104 (PMC11545822; doi:10.3390/healthcare12212104)
Supplement: Supplementary file 1 [file healthcare-12-02104-s001.zip › healthcare-3180664-supplementary.pdf]

Supplemental data

Efficacy of Horticultural Therapy on Positive, Negative and Affective Symptoms in Individuals with Schizophrenia: A Systematic Review and Meta-analysis of Randomized Controlled Trials

Yi-Wen Lee [1], Tzu-Ting Chen [2,3], Chih-Wei Hsu [2], Ming-De Chen [4], Pao-Yen Lin [2], Yu-Chi Huang [2], Chi-Fa Hung [2], Chyi-Rong Chen [2,3,*]

[1] Department of Nursing, Kaohsiung Chang Gung Memorial Hospital, College of Medicine, Chang Gung University, Kaohsiung 833401, Taiwan
[2] Department of Psychiatry, Kaohsiung Chang Gung Memorial Hospital, College of Medicine, Chang Gung University, Kaohsiung 833401, Taiwan
[3] Department of Occupational Therapy, Shu-Zen Junior College of Medicine and Management, Kaohsiung 821004, Taiwan
[4] Department of Occupational Therapy, College of Health Sciences, Kaohsiung Medical University, Kaohsiung City, Taiwan
* Correspondence: ccr776@cgmh.org.tw

Table of content

Content	Page
Table S1. Search texts and strategies	3
Table S2. Details of included studies	4-9
Table S3. Meta-regression for examining moderator relationships.	10-11
Table S4. Subgroup analysis based on initial severity of symptoms.	12-13
Table S5. GRADE summary table	14-15
Figure S1. Funnel plot of total symptom (short term effect)	16
Figure S2. Funnel plot of total symptom (long term effect)	16
Figure S3. Funnel plot of positive symptoms (short term effect)	17
Figure S4. Funnel plot of positive symptoms (long term effect)	17
Figure S5. Funnel plot of negative symptoms (short term effect)	18
Figure S6. Funnel plot of negative symptoms (long term effect)	18
Figure S7. Funnel plot of Depression (short term effect)	19
Figure S8. Funnel plot of Depression (long term effect)	19
Figure S9. Funnel plot of anxiety (short term effect)	20
PRISMA Checklist	21-24

Table S1. Search texts and strategies

Database	Search terms	Filters
PubMed	(Schizophrenia OR "Severe mental illness") AND (Gardening [Mesh] OR "Horticultural Therapy" [Mesh] OR horticultur* OR plant OR garden OR farm) AND (pressure OR depress* OR anxiety OR mood OR affect OR stress OR symptom)	Title/Abstract; Randomized controlled trial
Embase	('severe mental illness'/exp OR 'schizophrenia'/exp) AND ('gardening'/exp OR 'horticultural therapy'/exp OR horticultur* OR garden OR plant OR farm) AND (pressure OR depress* OR anxiety OR mood OR affect OR stress OR symptom)	Title/Abstract; Randomized controlled trial
Cochrane Library	(Schizophrenia OR (MeSH descriptor: [Schizophrenia] explode all trees) OR severe mental illness) AND ((MeSH descriptor: [Gardening] explode all trees) OR (MeSH descriptor: [Horticultural Therapy] explode all trees) OR horticultur* OR garden OR farm OR plant) AND (pressure OR depress* OR anxiety OR mood OR affect OR stress OR symptom)	Title/Abstract; Trials
CINAHL	(Schizophrenia OR (MH schizophrenia) OR severe mental illness) AND (gardening OR "Horticultural therapy" OR horticultur* OR farm OR plant) AND (stress OR affect OR mood OR anxiety OR depress* OR pressure OR symptom)	Abstract
CEPS	((([ALL]=(思覺失調) OR [ALL]=(精神分裂)) OR [ALL]=(精障)) AND ([ALL3]=(園藝) OR [ALL3]=(農)))	N/A
CNKI	(精神分裂 OR 精障) AND (農療 OR 園藝 OR 農)	Title/keywords
Wanfang	(精神分裂 OR 精障) AND (農療 OR 園藝 OR 農)	N/A
Yiigle	(精神分裂 OR 精障) AND (農療 OR 園藝 OR 農)	N/A

Table S2. Details of included studies

Study (location)	Sample Size (setting)	Age (mean ± SD)	Horticultural intervention	Control condition	Supervision/ Form	Outcome Measures *Primary	Duration of Intervention
Ban 2001 [51] (China)	HT: 19 CG: 19 (inpatients)	All: 39.07 ± 8.60	Flower planting, bonsai creation	Treatment as usual	Nurse/Group	BPRS	1 hour/session, 5 hours/week, last for 3 months
Cao 2013 [52] (China)	HT: 40 CG: 40 (inpatients)	HT: 41.2 ± 8.6 CG: 43.4 ± 9.4	Corn cultivation	Treatment as usual	Horticultural therapists/Group	PANSS	1.5 hours/session, 7.5 hours/week, and additional 1.5 hours lecture, last for 6 months
Chen 2013 [53] (China)	HT: 40 CG: 40 (inpatients)	HT: 43.26 ± 10.26 CG: 45.21 ± 9.87	Vegetable and fruit cultivation, horticultural crafts	Treatment as usual	Horticultural therapists/Group	PANSS	1-2 hours/day, 8-12 hours/week, and last for 2 years
Chen 2023 [54], Chen 2023 [55] (China)	HT: 63 CG: 61 (inpatients)	HT: 38.95 ± 12.83 CG: 41.23 ± 13.23	Plant cultivation	Treatment as usual	Nurse/Group	BPRS, HAMD	5 days/week, last for 6 weeks
Ding 2020 [56] (China)	HT: 31 CG: 31 (inpatients)	HT: 51.77 ± 7.24 CG: 52.22 ± 6.96	Bonsai creation	Treatment as usual	Rehabilitation therapist/Group	PANSS	1.5-2 hours/session, 1 session/week, last for 12 weeks
He 2020 [57] (China)	HT: 30 CG: 29 (inpatients)	HT: 44.20 ± 13.695 CG: 46.66 ± 13.401	Lettuce cultivation, sensory stimulation	Treatment as usual	Horticultural therapists/Group	BPRS	1 hour/session, 1 session/week, last for 6 weeks

Hu 2019 [58] (China)	HT: 58 CG: 58 (inpatients)	HT: 48 ± 8 CG: 48 ± 7	Plant cultivation, landscape viewing	Treatment as usual	Social worker/ Group	PANSS	1 hour/session, 2 sessions/week, last for 12 weeks
Huang 2017 [59] (China)	HT: 60 CG: 60 (inpatients)	All: 63.0 ± 8.60	Flower cultivation	Treatment as usual	Horticultural therapists/Group	BPRS	Last for 3 months. Not mentioned for other information.
Kam 2010 [82] (Hong Kong)	HT: 12 CG: 12 (shelter workshop)	HT: 45.3 ± 10.38 CG: 43.3 ± 11.7	Plant cultivation, landscape viewing	Treatment as usual	Occupational therapist/Group	DASS-21	1 hour/session, a total of 10 session within 2 weeks.
Kenmochi 2019 [85] (Japan)	HT: 11 CG: 12 (inpatients)	HT: 55.8 ± 7.5 CG: 53 ± 8.9	Vegetable cultivation and obsevation, eating	Occuptional therapy	Horticultural therapists/Group	PANSS	1 hour/session, 1 session/ week, last for 11 weeks.
Kong 2019 [60] (China)	HT:34 CG: 35 (community patients)	HT: 37.53 ± 11.24 CG: 37.09 ± 15.81	Plant cultivation, horticultural crafts	Treatment as usual	Rehabilitation therapist /Group	PANSS	1.5 hours/session, 1 session/week, last for 12 weeks.
Lee 2021 [84] (Taiwan)	HT:21 CG: 21 (daycare patients)	HT: 44.95 ± 11.05 CG: 49.00 ± 7.52	Plant cultivation	Treatment as usual	Occupational therapist and nurse/Group	DASS-21	80 minutes/session, 2 sessions/week, last for 4 weeks.
Lei 2019 [61] (China)	HT:47 CG: 47 (outpatients)	HT: 30.66 ± 7.96 CG: 31.32 ± 8.87	Plant cultivation	Treatment as usual	Horticultural therapists/Group	SANS	2 hours/session, 3-4 sessions/week, last for 12 months.

Li 2015 [62] (China)	HT:32 CG: 32 (inpatients)	HT: 43.51 ± 8.66 CG: 42.88 ± 8.49	Plant cultivation	Treatment as usual	Horticultural therapists/Group	NOSIE	Last for 6 months. Not mentioned for other information.
Li 2020 [63] (China)	HT:40 CG: 40 (inpatient)	HT: 57.65 ± 7.25 CG: 61.68 ± 6.43	Plant cultivation, cooking, landscape maintenance	Treatment as usual	Mental health professionals /Group	PANSS	0.5 hour/session, 5 sessions/week, last for 12 months.
Liang 2022 [64] (China)	HT:66 CG: 66 (inpatients)	HT: 37.65 ± 6.73 CG: 37.86 ± 6.94	Flower and vegetable cultivation, bonsai creation, horticultural craft	Treatment as usual	Nurse/Group	PANSS	Last for 6 months. Not mentioned for other information.
Liu 2018 [67] (China)	HT:30 CG: 30 (inpatients)	HT: 46.4 ± 8.5 CG: 46.5 ± 8.2	Flower and vegetable cultivation	Treatment as usual	Mental health professionals /Group	PANSS	1-1.5 hours/week, 5-8 hours/week, last for 24 weeks.
Liu 2021 [66] (China)	HT:86 CG: 86 (inpatients)	HT: 36.61 ± 8.24 CG: 37.16 ± 8.15	Plant cultivation, bonsai creation, horticultural craft, landscape viewing	Treatment as usual	Horticultural therapists/Group	BPRS, SAD	Last for 3 months. Not mentioned for other information.
Liu 2023 [65] (China)	HT:50 CG: 50 (inpatients)	HT: 44.52 ± 9.36 CG: 44.39 ± 9.51	Plant cultivation, horticultural craft	Treatment as usual	Nurse/Group	PANSS	Last for 3 months. Not mentioned for other information.

Lu 2010 [68] (China)	HT:34<br>CG: 34<br>(inpatients)	HT: 42 ± 12<br>CG: 40 ± 11	Vegetable, fruit and flower cultivation	Treatment as usual	Horticultural therapists/Group	PANSS	1 hour/session,<br>3-4 seesions/week,<br>with additional 1 hour lecture session once biweekly,<br>last for 12 months.
Shi 2020 [69] (China)	HT:30<br>CG: 30<br>(inpatients)	HT: 34.32 ± 1.15<br>CG: 34.30 ± 1.16	Plant cultivation	Treatment as usual	Horticultural therapists/Group	SANS	2.5 hours/session, 3-4 sessions/week, last for 3 months.
Siu 2020 [81] (Hong Kong)	HT:37<br>CG: 36<br>(shelter workshop)	HT: 50.8 ± 10.5<br>CG: 49.7 ± 8.7	Plant cultivation, horticultural craft	Treatment as usual	Horticultural therapists/Group	DASS-21	1.25 hours/session, with a total of 8 sessions.
Sun 2024 [80] (China)	HT:40<br>CG: 40<br>(inpatients)	HT: 48.5 ± 8.8<br>CG: 46.0 ± 8.0	Plant cultivation	Treatment as usual	Mental health professionals /Group	HAMD,<br>HAMA	1 hour/session,<br>3 hours/week,<br>last for 24 weeks.
Tao 2017 [70] (China)	HT:90<br>CG: 90<br>(inpatients)	HT: 41.5 ± 6.8<br>CG: 40.4 ± 7.5	Vegetable and fruit cultivation	Treatment as usual	Nurse/Group	SANS	1-2 hours/session,<br>5-8 hours/week,<br>last for 24 weeks.
Wang 2022 [71] (China)	HT:50<br>CG: 50<br>(inpatients)	HT: 38.19 ± 5.40<br>CG: 37.32 ± 7.20	Vegetable, fruit and flower cultivation	Treatment as usual	Mental health professionals /Group	PANSS	1-1.5 hours/session,<br>6-8 hours/week,<br>last for 24 weeks.
Yang 2009 [73] (China)	HT:34<br>CG: 36<br>(inpatients)	HT: 38.72 ± 11.46<br>CG: 39.47 ±	Horticultural activities	Treatment as usual	Rehabilitation therapis	BPRS	2 hours/sessions,<br>3 sessions/week, with additional 2 hours of

		10.52			t /Group		lecture, last for 24 weeks.
Yang 2011 [72] (China)	HT:49 CG: 49 (inpatients)	All: 44.7 ± 1.08	Plant cultivation	Treatment as usual	Nurse/Group	SANS	1 hour/session, 6-8 hours/week, last for 12 months.
Yang 2017 [83] (Taiwan)	HT:56 CG: 49 (inpatients)	HT: 49.56 ± 9.155 CG: 48.09 ± 9.171	Plant cultivation, bonsai creation, horticultural craft	Occupational therapy	Nurse/Group	STAI	1-1.5 hours/session, 1 session/week, last for 8 weeks.
Yin 2015 [74] (China)	HT:18 CG: 18 (inpatients)	HT: 40 ± 8.5 CG: 40.2 ± 8.2	Vegetable and fruit cultivation	Treatment as usual	Mental health professionals /Group	SANS	2 hours/session, 5-6 sessions/week, last for 10 months.
Zhang 2015 [75] (China)	HT:45 CG: 38 (inpatient)	HT: 42.52 ± 9.25 CG: 43.26 ± 8.91	Plant cultivation	Treatment as usual	Rehabilitation therapist /Group	NOSIE	2 hours/session, 3-4 sessions/week, last for 6 months.
Zhao 2022 [76] (China)	HT:45 CG: 45 (inpatients)	HT: 49.51 ± 7.62 CG: 49.96 ± 9.52	Plant cultivation	Treatment as usual	Nurse/Group	PANSS	2 hours/session, 5 sessions/week, last for 12 months.
Zhou 2003 [77] (China)	HT:33 CG: 31 (inpatients)	HT: 32.94 ± 9.27 CG: 30.17 ± 8.29	Vegetable, fruit and flower cultivation	Treatment as usual	Rehabilitation therapist /Group	STAI	3.5 hours/session, 5 sessions/week, last for 1 month.
Zhu 2016 [78] (China)	HT:55 CG: 55 (inpatients)	All: 46.5 ± 9.0	Plant cultivation	Treatment as usual	Rehabilitation therapist/Group	PANSS	1.5 hours/sesssion, 3 sessions/week, last for 12 weeks.

Zhu 2019 [79] (China)	HT:70 CG: 70 (inpatients)	HT: 46.97 ± 11.48 CG: 46.96 ± 9.54	Plant cultivation, food making	Treatment as usual	Rehabilitation therapist/Group	PANSS, NOSIE	1-1.5 hours/session, 5-7 hours/week, last for 12 weeks

HT, Horticultural Therapy; CG, Control Group; BPRS, Brief Psychiatric Rating Scale; PANSS, Positive and Negative Symptoms Scale; HAMD, Hamilton depression scale; HAMA, Hamilton anxiety scale; DASS-21, Depression Anxiety Stress Scales-21; SANS, Scale for the Assessment of Negative Symptoms; NOSIE, Nurses' Observation for Inpatient Evaluation; SAD, Social Avoidance and Distress Scale; STAI, State-Trait Anxiety Inventory.

Table S3. Meta-regression for examining moderator relationships.

Outcome	Moderator	Studies	β	p value
Short term effect				
Total symptoms	Mean age	11	0.013	.397
	Female percentage	11	-0.003	.592
	Illness years	10	-0.018	.240
	Weekly minutes	7	0.005	.660
Positive symptoms	Mean age	5	0.021	.758
	Female percentage	5	0.024	.460
	Illness years	5	-0.041	.353
	Weekly minutes	5	-0.001	.947
Negative symptoms	Mean age	8	0.013	.614
	Female percentage	7	0.008	.672
	Illness years	8	-0.016	.515
	Weekly minutes	8	-0.001	.637
Depression	Mean age	8	0.019	.613
	Female percentage	8	-0.002	.974
	Illness years	7	-0.008	.800
	Weekly minutes	6	0.001	.714
Anxiety	Mean age	9	-0.020	.301
	Female percentage	9	-0.008	.402
	**Illness years**	**8**	**-0.036**	**.005***
	Weekly minutes	7	0.000	.550
Long term effect*				
Total symptoms	Mean age	10	-0.040	.266
	Female percentage	9	-0.052	.130
	Illness years	10	-0.020	.545
	**Weekly minutes**	**9**	**0.004**	**.031***
Positive symptoms	Mean age	7	-0.027	.483
	Female percentage	6	0.012	.752
	Illness years	7	-0.052	.080
	Weekly minutes	6	-0.000	.941
Negative symptoms	Mean age	11	-0.023	.486
	Female percentage	8	-0.035	.247
	Illness years	11	-0.011	.705
	**Weekly minutes**	**10**	**0.004**	**.018***

*There were insufficient data on the long-term effect of depression and anxiety for meta-regression.

Table S4. Subgroup analysis based on initial severity of symptoms.

				Effect size		Heterogeneity	
**Short term effect (≦3 months)**	**Studies**	**Total n**	**SMD**	**95% CI**	***p* value**	***Q* value**	***p* value**
**Total symptoms**							
Mildly illness	7	576	0.689	0.390 – 0.988	<0.001		
Moderately illness	2	162	0.594	0.279 – 0.909	<0.001	0.304	0.859
Severely illness	2	296	0.787	0.008 – 1.567	0.048		
**Positive symptoms**							
Mildly illness	4	312	0.360	0.121 – 0.600	0.003		
Moderately illness	1	100	1.968	1.490 – 2.445	<0.001	34.756	<0.001
Severely illness	NA	NA	NA	NA	NA		
**Negative symptoms**							
Mildly illness	4	318	0.661	0.435 – 0.887	<0.001		
Moderately illness	NA	NA	NA	NA	NA	11.365	0.001
Severely illness	1	100	1.520	1.075 – 1.965	<0.001		
**Depression**							
Mildly illness	3	208	0.311	-0.169 – 0.790	0.202		
Moderately illness	NA	NA	NA	NA	NA	5.957	0.015
Severely illness	1	124	1.070	0.694 – 1.446	<0.001		
**Anxiety**							
Mildly illness	2	139	0.598	-0.310 – 1.507	0.197		
Moderately illness	NA	NA	NA	NA	NA	0.235	0.628
Severely illness	1	172	0.836	0.524 – 1.147	<0.001		
				**Effect size**		**Heterogeneity**	
**Long-term effect (>3 months)**	**Studies**	**Total n**	**SMD**	**95% CI**	***p* value**	***Q* value**	***p* value**
**Total symptoms**							

Mildly illness	4	308	0.686	-0.052 – 1.425	0.069		
Moderately illness	6	592	1.858	1.258 – 2.458	<0.001	5.829	0.016
Severely illness	NA	NA	NA	NA	NA		
**Positive symptoms**							
Mildly illness	2	170	0.116	-0.185 – 0.418	0.449		
Moderately illness	5	512	0.900	0.085 – 1.716	0.031	3.121	0.077
Severely illness	NA	NA	NA	NA	NA		
**Negative symptoms**							
Mildly illness	2	170	1.155	-0.841 – 3.151	0.257		
Moderately illness	5	512	1.331	0.701 – 1.961	<0.001	0.027	0.869
Severely illness	NA	NA	NA	NA	NA		
**Depression**							
Mildly illness	1	83	0.145	-0.287 – 0.577	0.511		
Moderately illness	1	140	0.389	0.055- 0.723	0.023	0.765	0.382
Severely illness	NA	NA	NA	NA	NA		
**Anxiety**							
Mildly illness	NA	NA	NA	NA	NA		
Moderately illness	NA	NA	NA	NA	NA	NA	NA
Severely illness	NA	NA	NA	NA	NA		

Table S5. GRADE summary table

Horticultural therapy compared to control for schizophrenia					
Outcomes	No of participants (studies) Follow-up	Certainty of the evidence (GRADE)	Relative Effect (95% CI)	Anticipated absolute effects	
				Risk with control	Risk difference with Horticultural therapy
Changes in total symptom in short term (≦3 months)	(11 RCTs)	⊕⊕○○	-	-	SMD 0.690 SD higher (0.463 higher to 0.916 higher)
Changes in total symptom in long term (＞3 months)	(10 RCTs)	⊕⊕○○	-	-	SMD 1.393 SD higher (0.858 higher to 1.928 higher)
Changes in positive symptom in short term (≦ 3 months)	(5 RCTs)	⊕○○○	-	-	SMD 0.695 SD higher (0.038 higher to 1.351 higher)
Changes in positive symptom in long term (＞ 3 months)	(7 RCTs)	⊕⊕○○	-	-	SMD 0.667 SD higher (0.077 higher to 1.258 higher)
Changes in negative symptom in short term (≦ 3 months)	(8 RCTs)	⊕⊕○○	-	-	SMD 0.681 SD higher (0.395 higher to 0.967 higher)
Changes in negative symptom in long term (＞ 3 months)	(11 RCTs)	⊕⊕○○	-	-	SMD 1.389 SD higher (0.935 higher to 1.842 higher)
Changes in depression in short term (≦ 3 months)	(8 RCTs)	⊕⊕○○	-	-	SMD 0.646 SD higher (0.334 higher to 0.959

					higher)
Changes in depression in long term (＞3 months)	(4 RCTs)	⊕○○○	-	-	SMD 0.707 SD higher<br>(0.198 higher to 1.217 higher)
Changes in anxiety in short term (≦ 3 months)	(9 RCTs)	⊕⊕○○	-	-	SMD 0.627 SD higher<br>(0.364 higher to 0.890 higher)
Changes in anxiety in short term (＞ 3 months)	(1 RCT)	⊕○○○	-	-	SMD 1.541 SD higher<br>(1.042 higher to 2.040 higher)

*The risk in the intervention group (and its 95% confidence interval) is based on the assumed risk in the comparison group and the relative effect of the intervention (and its 95% CI).

CI: confidence interval; SMD: standardized mean difference

GRADE Working Group grades of evidence

High certainty: we are very confident that the true effect lies close to that of the estimate of the effect.

Moderate certainty: we are moderately confident in the effect estimate: the true effect is likely to be close to the estimate of the effect, but there is a possibility that it is substantially different.

Low certainty: our confidence in the effect estimate is limited: the true effect may be substantially different from the estimate of the effect.

Very low certainty: we have very little confidence in the effect estimate: the true effect is likely to be substantially different from the estimate of effect.

Figure S1. Funnel plot of total symptom (short term effect) (Egger's *p*=.605)

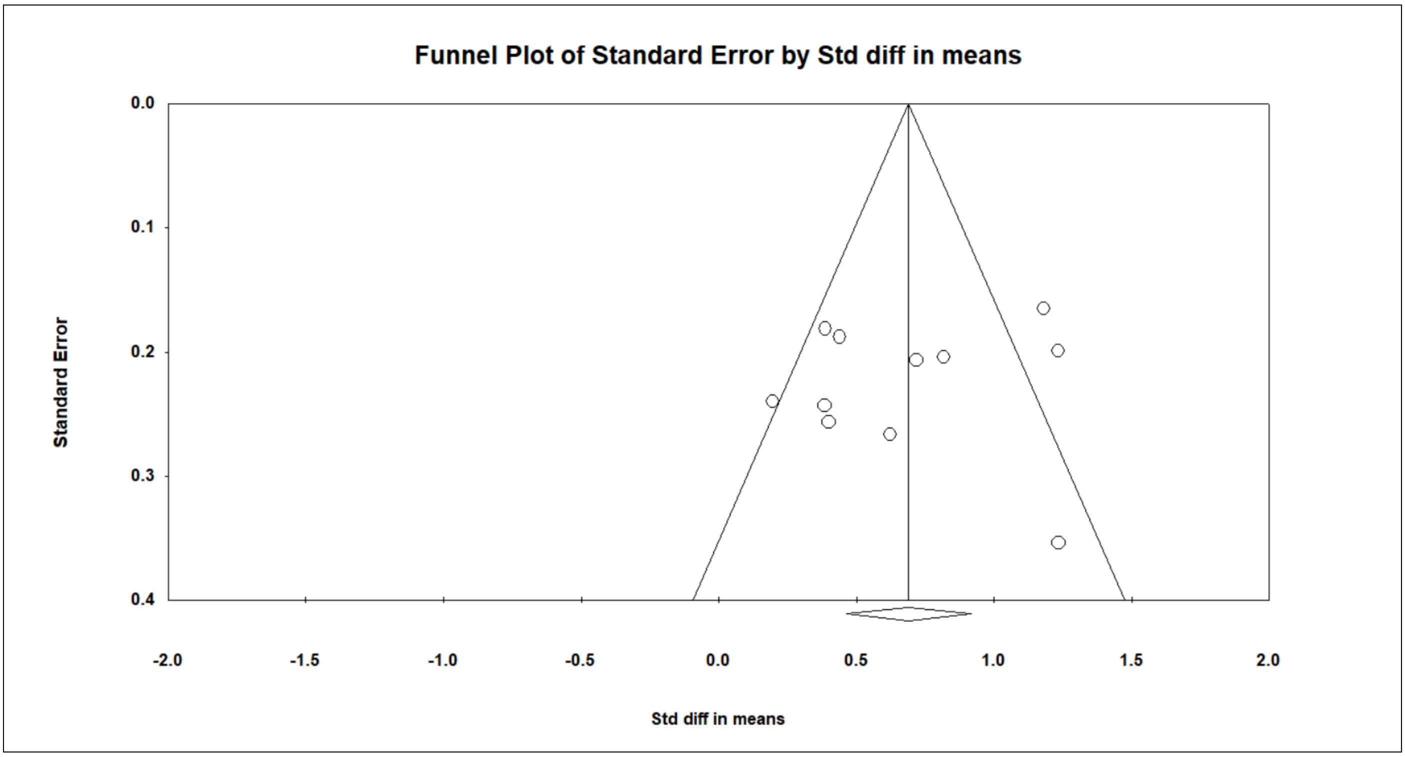

Figure S2. Funnel plot of total symptom (long term effect) (Egger's *p*=.134)

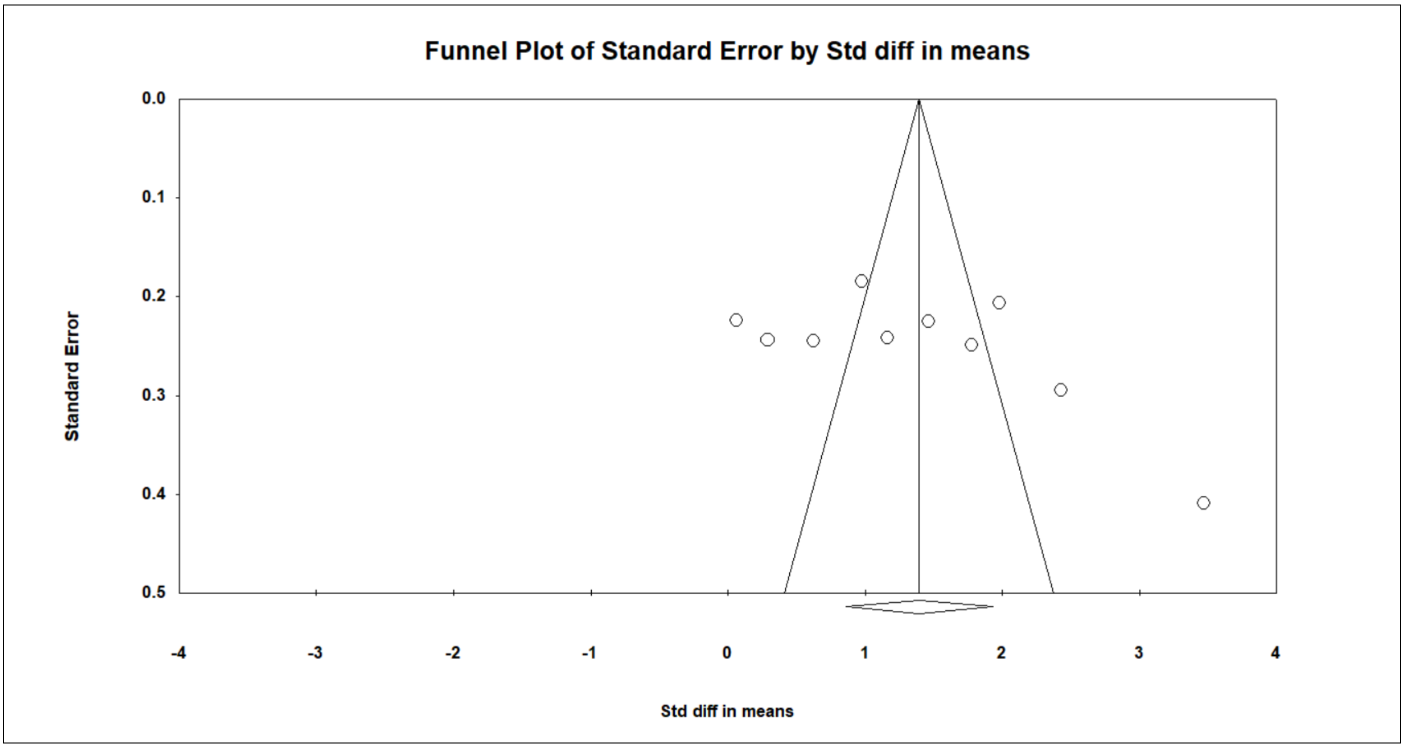

Figure S3. Funnel plot of positive symptoms (short term effect) (Egger's *p*=.801)

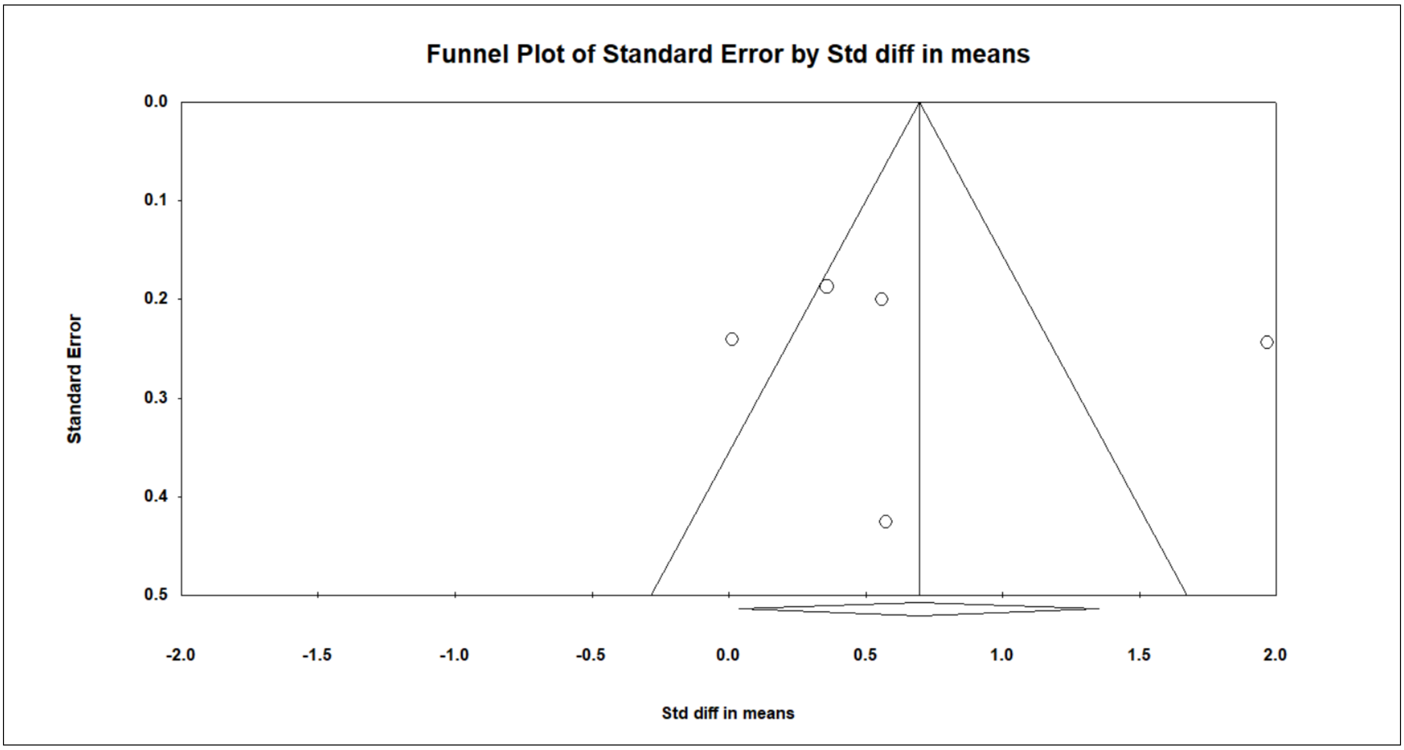

Figure S4. Funnel plot of positive symptoms (long term effect) (Egger's *p*=.070)

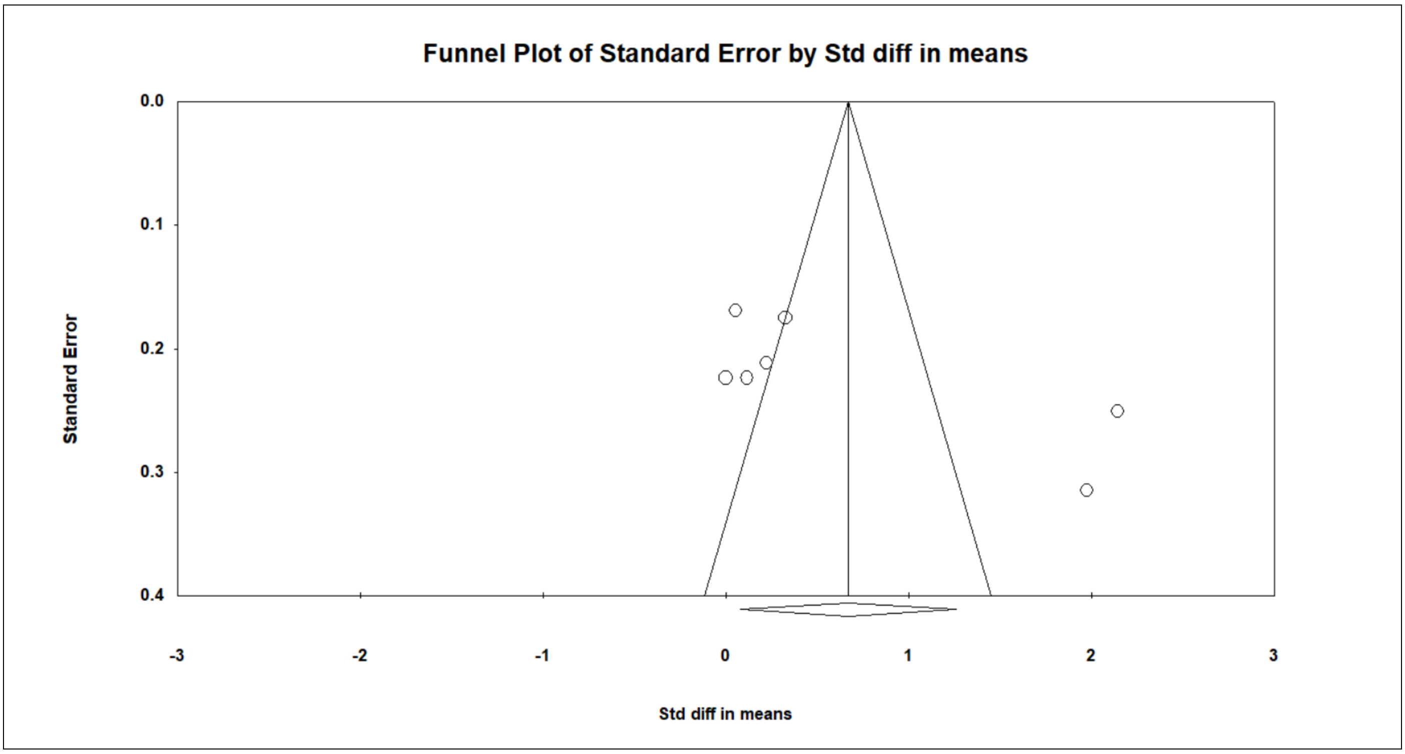

Figure S5. Funnel plot of negative symptoms (short term effect) (Egger's *p*=.873)

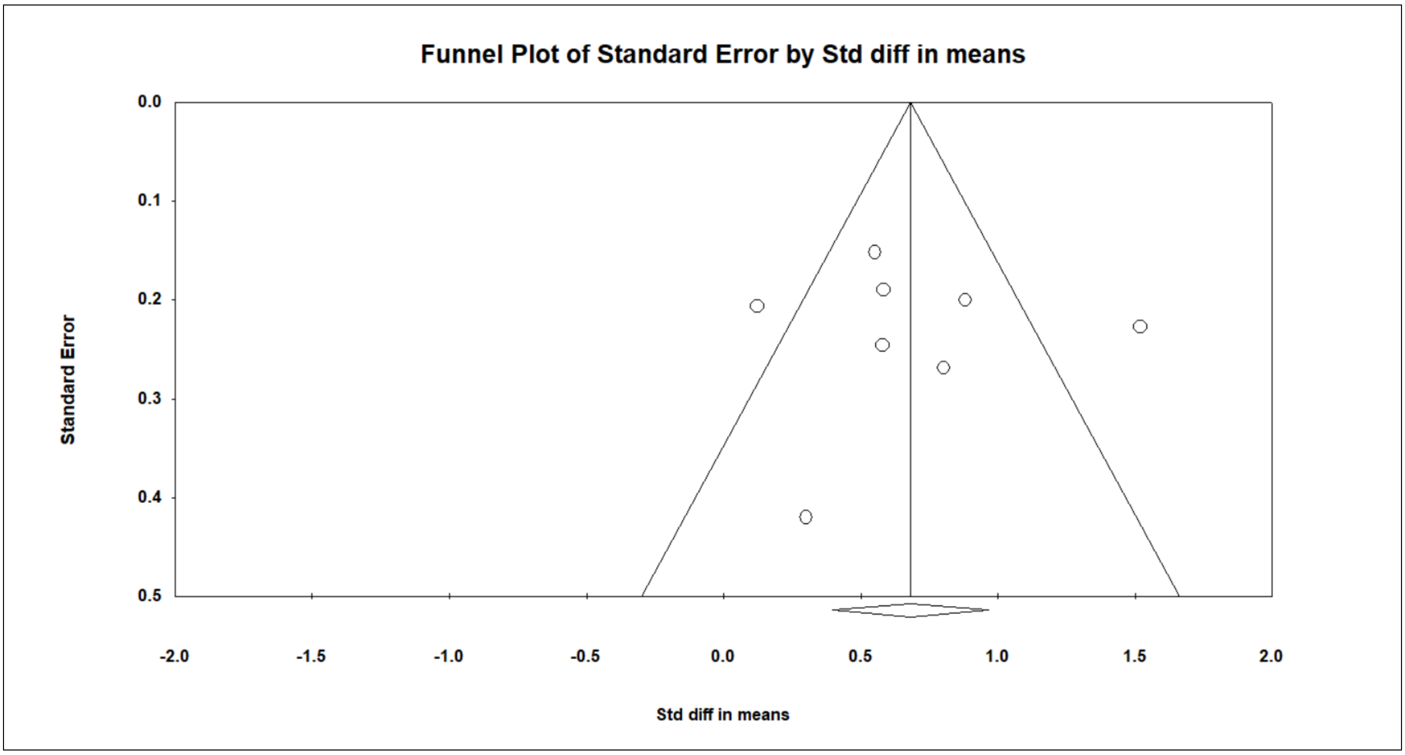

Figure S6. Funnel plot of negative symptoms (long term effect) (Egger's *p*=.210)

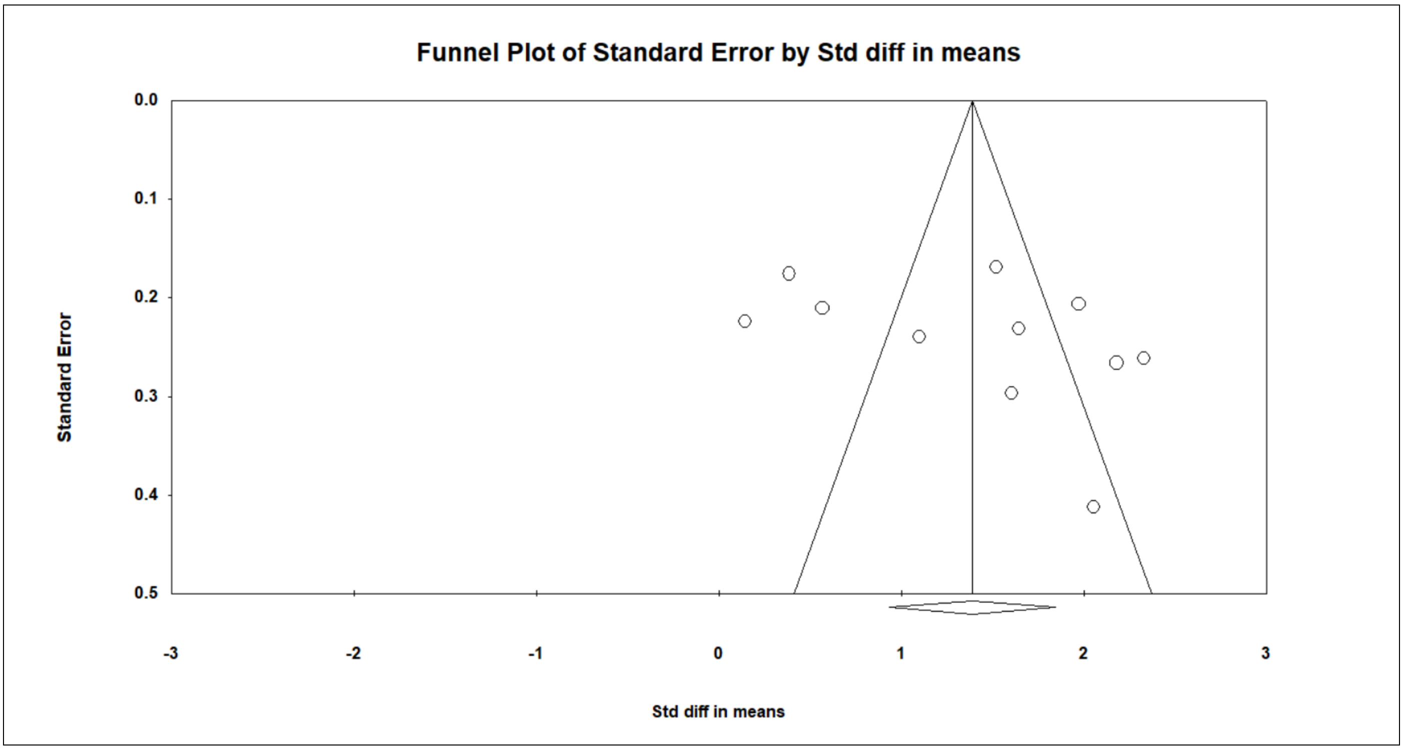

Figure S7. Funnel plot of Depression (short term effect) (Egger's *p*=.451)

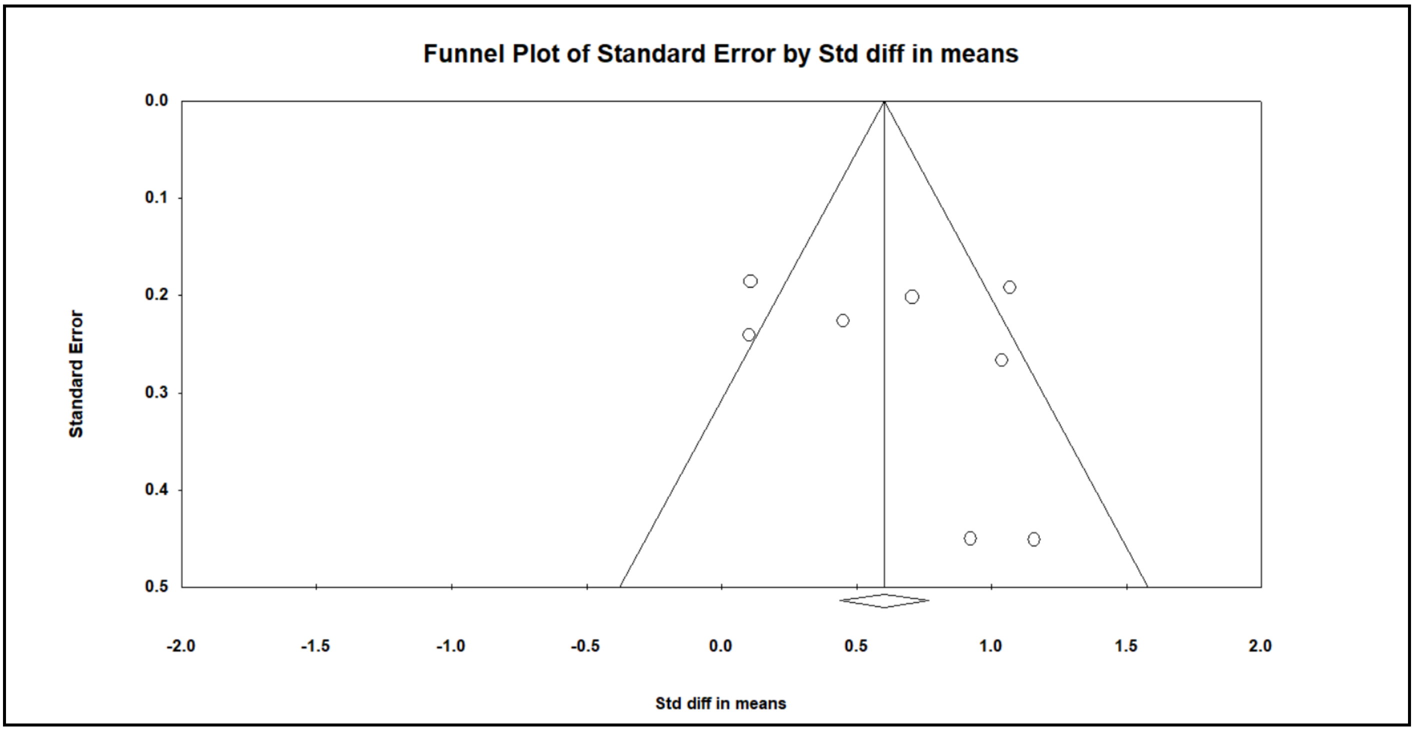

Figure S8. Funnel plot of Depression (long term effect) (Egger's *p*=.296)

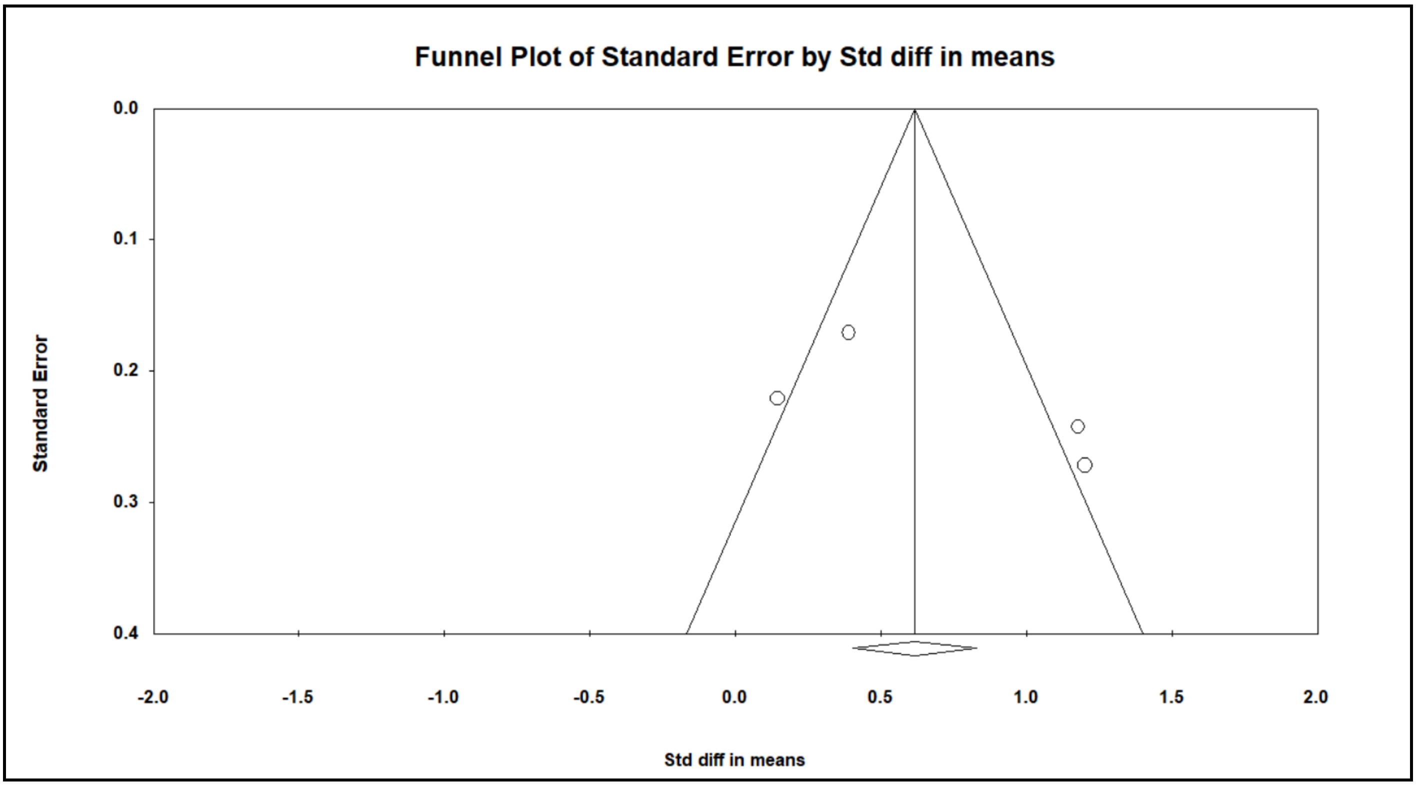

Figure S9. Funnel plot of anxiety (short term effect) (Egger's *p*=.231)

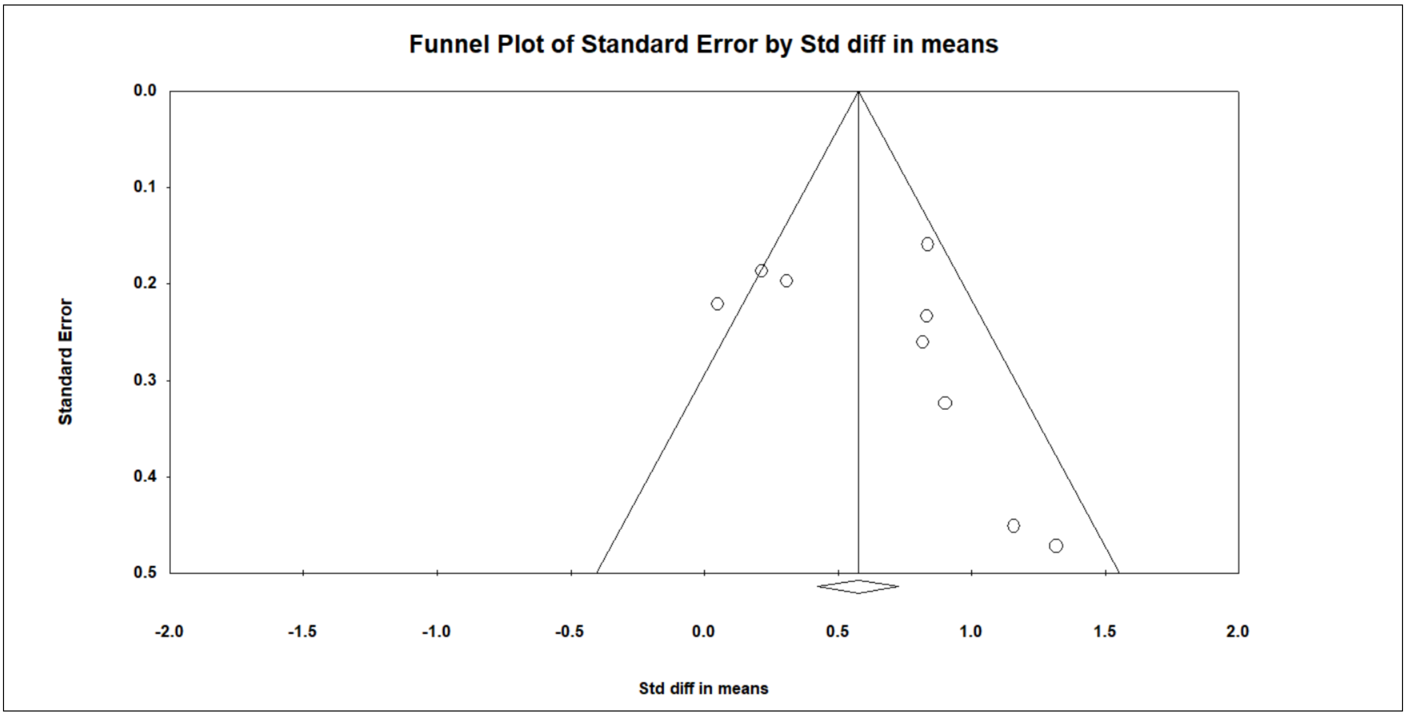

PRISMA Checklist

Section and Topic	Item #	Checklist item	Location where item is reported
**TITLE**			
Title	1	Identify the report as a systematic review.	Title page
**ABSTRACT**			
Abstract	2	See the PRISMA 2020 for Abstracts checklist.	abstract
**INTRODUCTION**			
Rationale	3	Describe the rationale for the review in the context of existing knowledge.	1-3
Objectives	4	Provide an explicit statement of the objective(s) or question(s) the review addresses.	3
**METHODS**			
Eligibility criteria	5	Specify the inclusion and exclusion criteria for the review and how studies were grouped for the syntheses.	4
Information sources	6	Specify all databases, registers, websites, organisations, reference lists and other sources searched or consulted to identify studies. Specify the date when each source was last searched or consulted.	3-4
Search strategy	7	Present the full search strategies for all databases, registers and websites, including any filters and limits used.	3-4, Table S1
Selection process	8	Specify the methods used to decide whether a study met the inclusion criteria of the review, including how many reviewers screened each record and each report retrieved, whether they worked independently, and if applicable, details of automation tools used in the process.	4
Data collection process	9	Specify the methods used to collect data from reports, including how many reviewers collected data from each report, whether they worked independently, any processes for obtaining or confirming data from study investigators, and if applicable, details of automation tools used in the process.	4
Data items	10a	List and define all outcomes for which data were sought. Specify whether all results that were compatible with each outcome domain in each study were sought (e.g. for all measures, time points, analyses), and if not, the methods used to decide which results to collect.	4
	10b	List and define all other variables for which data were sought (e.g. participant and intervention characteristics, funding sources). Describe any	4

Section and Topic	Item #	Checklist item	Location where item is reported
		assumptions made about any missing or unclear information.	
Study risk of bias assessment	11	Specify the methods used to assess risk of bias in the included studies, including details of the tool(s) used, how many reviewers assessed each study and whether they worked independently, and if applicable, details of automation tools used in the process.	4
Effect measures	12	Specify for each outcome the effect measure(s) (e.g. risk ratio, mean difference) used in the synthesis or presentation of results.	4
Synthesis methods	13a	Describe the processes used to decide which studies were eligible for each synthesis (e.g. tabulating the study intervention characteristics and comparing against the planned groups for each synthesis (item #5)).	4
	13b	Describe any methods required to prepare the data for presentation or synthesis, such as handling of missing summary statistics, or data conversions.	4-5
	13c	Describe any methods used to tabulate or visually display results of individual studies and syntheses.	4-5
	13d	Describe any methods used to synthesize results and provide a rationale for the choice(s). If meta-analysis was performed, describe the model(s), method(s) to identify the presence and extent of statistical heterogeneity, and software package(s) used.	4-5
	13e	Describe any methods used to explore possible causes of heterogeneity among study results (e.g. subgroup analysis, meta-regression).	4-5
	13f	Describe any sensitivity analyses conducted to assess robustness of the synthesized results.	4-5
Reporting bias assessment	14	Describe any methods used to assess risk of bias due to missing results in a synthesis (arising from reporting biases).	4
Certainty assessment	15	Describe any methods used to assess certainty (or confidence) in the body of evidence for an outcome.	6
**RESULTS**			
Study selection	16a	Describe the results of the search and selection process, from the number of records identified in the search to the number of studies included in the review, ideally using a flow diagram.	5, Figure 1

Section and Topic	Item #	Checklist item	Location where item is reported
	16b	Cite studies that might appear to meet the inclusion criteria, but which were excluded, and explain why they were excluded.	Figure 1
Study characteristics	17	Cite each included study and present its characteristics.	5, Table S2
Risk of bias in studies	18	Present assessments of risk of bias for each included study.	6-7, Figure 2
Results of individual studies	19	For all outcomes, present, for each study: (a) summary statistics for each group (where appropriate) and (b) an effect estimate and its precision (e.g. confidence/credible interval), ideally using structured tables or plots.	7-12, Figures 3-7, Table 1
Results of syntheses	20a	For each synthesis, briefly summarise the characteristics and risk of bias among contributing studies.	6, Table S2
	20b	Present results of all statistical syntheses conducted. If meta-analysis was done, present for each the summary estimate and its precision (e.g. confidence/credible interval) and measures of statistical heterogeneity. If comparing groups, describe the direction of the effect.	7-12, Figures 3-7, Table 1
	20c	Present results of all investigations of possible causes of heterogeneity among study results.	12-13, Table S3-S4
	20d	Present results of all sensitivity analyses conducted to assess the robustness of the synthesized results.	10
Reporting biases	21	Present assessments of risk of bias due to missing results (arising from reporting biases) for each synthesis assessed.	6, Figure 2
Certainty of evidence	22	Present assessments of certainty (or confidence) in the body of evidence for each outcome assessed.	13, Table S5
**DISCUSSION**			

Section and Topic	Item #	Checklist item	Location where item is reported
Discussion	23a	Provide a general interpretation of the results in the context of other evidence.	13
	23b	Discuss any limitations of the evidence included in the review.	14-15
	23c	Discuss any limitations of the review processes used.	14-15
	23d	Discuss implications of the results for practice, policy, and future research.	15
**OTHER INFORMATION**			
Registration and protocol	24a	Provide registration information for the review, including register name and registration number, or state that the review was not registered.	5
	24b	Indicate where the review protocol can be accessed, or state that a protocol was not prepared.	5
	24c	Describe and explain any amendments to information provided at registration or in the protocol.	NA
Support	25	Describe sources of financial or non-financial support for the review, and the role of the funders or sponsors in the review.	15-16
Competing interests	26	Declare any competing interests of review authors.	15-16
Availability of data, code and other materials	27	Report which of the following are publicly available and where they can be found: template data collection forms; data extracted from included studies; data used for all analyses; analytic code; any other materials used in the review.	15-16

From: Page MJ, McKenzie JE, Bossuyt PM, Boutron I, Hoffmann TC, Mulrow CD, et al. The PRISMA 2020 statement: an updated guideline for reporting systematic reviews. BMJ 2021;372:n71. doi: 10.1136/bmj.n71

For more information, visit: http://www.prisma-statement.org/
